# Multi‐state Photoswitching in Thienoquinoid‐Based Fluorescent Trithiophenes

**DOI:** 10.1002/chem.71423

**Published:** 2026-07-17

**Authors:** Rio Nishimura, Stefan Hecht, Shohei Saito, Ken‐ichi Yamashita

**Affiliations:** ^1^ Department of Chemistry Graduate School of Science The University of Osaka Toyonaka Osaka Japan; ^2^ Department of Chemistry Humboldt‐Universität zu Berlin Berlin Germany; ^3^ Center for the Science of Materials Berlin Humboldt‐Universität zu Berlin Berlin Germany; ^4^ Innovative Catalysis Science Division Institute for Open and Transdisciplinary Research Initiatives (ICS‐OTRI) The University of Osaka Suita Osaka Japan

**Keywords:** conjugation, fluorescence, geometrical isomer, photoisomerization, thienoquinoid

## Abstract

Achieving multi‐site photoisomerization within a single π‐conjugated scaffold while retaining luminescent functionality remains a formidable challenge, as electronic coupling between isomerizable units typically promotes intramolecular energy transfer that suppresses sequential switching, and the additional nonradiative pathways inherent to multiple isomerizable bonds further impede emissive behavior. Herein, we report a methine‐bridged trithiophene bearing 2,6‐dichlorophenyl substituents, in which two C═C double bonds embedded in a thienoquinoid framework undergo photoisomerization to afford three interconvertible geometrical isomers (*ZZ*, *EZ*, and *EE*). The steric constraint imposed by the *ortho*‐chloro substituents suppresses nonradiative torsional relaxation, yielding a fluorescence quantum yield of 0.40 for the *ZZ*‐isomer in cyclohexane, which decreases upon photoisomerization to the *EZ*‐ and *EE*‐isomers—a significant enhancement over the corresponding derivative lacking *ortho*‐substituents on the aryl groups, accompanied by a markedly reduced Stokes shift. The photostationary state composition varies systematically with the irradiation wavelength, primarily reflecting differences in the molar absorption coefficients of the three isomers, thereby enabling wavelength‐selective multi‐state control over isomer distributions inaccessible under thermodynamic equilibrium. Fluorescence lifetime measurements revealed isomer‐dependent excited‐state lifetimes, confirming that molecular geometry governs the photophysical properties within this conjugated framework. This study establishes a molecular design strategy for luminescent multi‐state photoswitches applicable to multi‐level optical information processing.

## Graphical abstract

A methine‐bridged trithiophene with a thienoquinoid core undergoes photoisomerization of both C═C bonds, affording three geometrical isomers in a ratio controlled by the irradiation wavelength. Despite full π‐conjugation, the system retains fluorescence, enabling wavelength‐addressable multi‐state switching with luminescent readout.

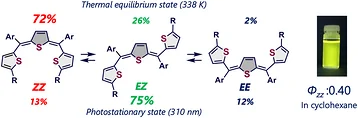

## 1 Introduction

Molecular photoswitches that access more than two distinct states offer enhanced functionality for molecular motors [1, 2], information‐storage materials [3, 4, 5], chemical computing [6, 7, 8, 9], and photoresponsive systems [10], and extend beyond the scope of conventional binary switching. When multiple photoisomerizable units are connected via electronically insulating linkers [11], each unit operates independently. In contrast, direct integration within a single π‐conjugated framework can generate an expanded set of metastable states through electronic coupling [12]. This principle has been exploited in third‐generation molecular motors reported by Feringa and coworkers [13, 14, 15, 16, 17], where the hybridization of motor scaffolds produces multiple metastable states, and in a dihydropyrene dimer developed by the Hecht group [18], in which photoisomerization at one site promotes switching at the other through cooperative electronic effects.

However, intramolecular energy transfer in electronically coupled architectures typically suppresses sequential isomerization [19]. In π‐conjugated diarylethene dimers, ring closure at one site inhibits the photoreaction at the other, and the second ring‐closure process has been reported to be achieved only through a redox process [20]. Although considerable progress has been made in combining fluorescence with single‐site photoisomerization [21, 22, 23, 24, 25, 26, 27, 28, 29, 30], extending this approach to multi‐site systems poses a distinct challenge: the additional nonradiative pathways associated with multiple isomerizable bonds make the retention of emissive character considerably more difficult. To date, the combination of multi‐site *E*/*Z* photoisomerization with fluorescence has been reported in dicyanodistyrylbenzene‐based systems [31] and in a hemipiperazine‐based switch [32].

Thienoquinoid motifs are prominent building blocks of organic semiconductors. Takimiya and coworkers reported that such thienoquinoid scaffolds undergo C═C geometrical isomerization even under ambient light, a property that has been regarded as a problem for device applications rather than being considered an opportunity for photoswitching [33]. We recently reported that methine‐bridged trithiophenes **1a** incorporating a thienoquinoid core exist as three isolable geometrical isomers (*ZZ*, *EZ*, and *EE*), whose relative thermal stabilities are governed by intramolecular S···S chalcogen bonding (Figure 1) [34]. Our preliminary observations further indicated that compound **1a** undergoes photoisomerization and exhibits weak fluorescence in solution (*Φ*_fl_ < 0.01), suggesting that appropriate structural modification could substantially enhance the emission while preserving the multi‐photoisomerizable character of the scaffold.

**FIGURE 1:**
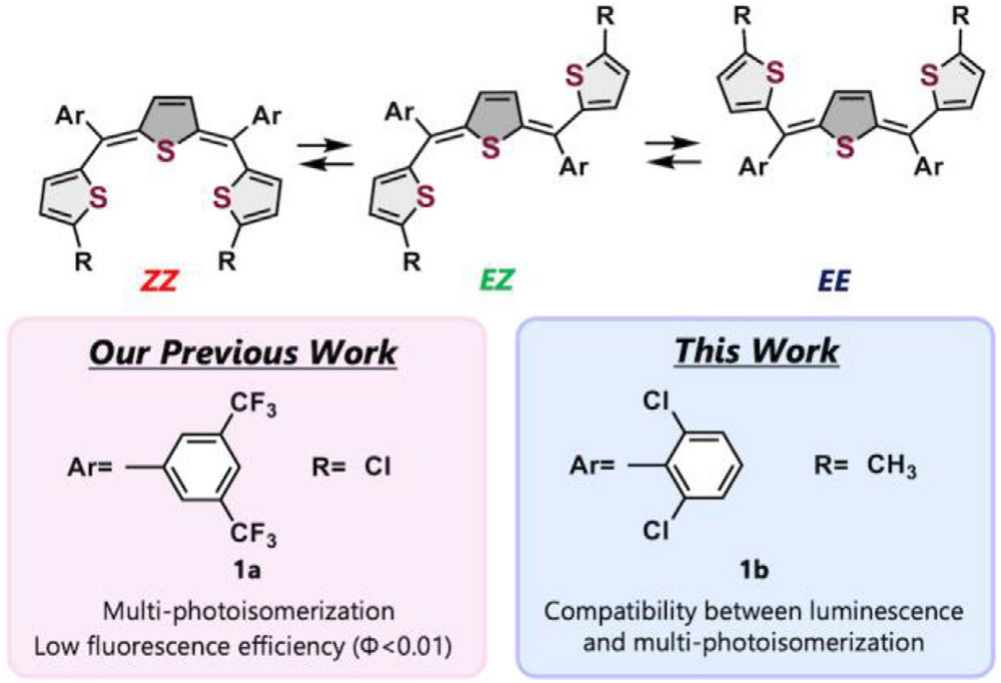
Methine‐bridged trithiophenes **1a** and **1b** with a thienoquinoid core, and the three geometrical isomers (*ZZ*, *EZ*, *EE*) arising from the *E*/*Z* configuration of the two C═C bonds.

In this study, we report the synthesis of methine‐bridged trithiophene derivative **1b** bearing 2,6‐dichlorophenyl substituents (Figure 1). Despite full electronic conjugation across the scaffold, both C═C double bonds underwent photoisomerization, affording access to three interconvertible geometrical isomers. As a result, the photostationary state (PSS) composition could be modulated by varying the irradiation wavelength, thereby demonstrating wavelength‐selective multi‐state optical control. Importantly, the system simultaneously retains significant fluorescence (*Φ*_fl_ = 0.40 for the *ZZ*‐isomer in cyclohexane), representing a rare example of a luminescent multi‐photoisomerizable molecule within a single π‐conjugated framework.

## 2 Results and Discussion

The molecular design of **1b** was guided by the observation that **1a** undergoes photoisomerization but exhibits only weak fluorescence in solution (*Φ*_fl_ < 0.01, Figures S17, S24, and Table S2). We hypothesized that nonradiative decay through the torsional motion of the terminal aryl groups is a major contributor to this weak emission. To test this hypothesis, 2,6‐dichlorophenyl groups were introduced at the methine carbons to produce **1b**. The two *ortho*‐chloro substituents were expected to restrict dihedral rotation and enforce a perpendicular arrangement of the aryl moieties relative to the thienoquinoid plane, thereby impeding the nonradiative decay pathway associated with large‐amplitude torsional motion.

Compound **1b** was synthesized via acid‐catalyzed condensation followed by oxidation in a manner analogous to that reported for **1a** [34]. It was characterized by ^1^H NMR, ^13^C NMR, and elemental analysis (see Supporting Information). Similar to **1a**, the crude product was obtained as a mixture of three geometrical isomers. The *ZZ*‐isomer of **1b** was isolated in pure form by recrystallization from dichloromethane/methanol (Figure 2a).

**FIGURE 2:**
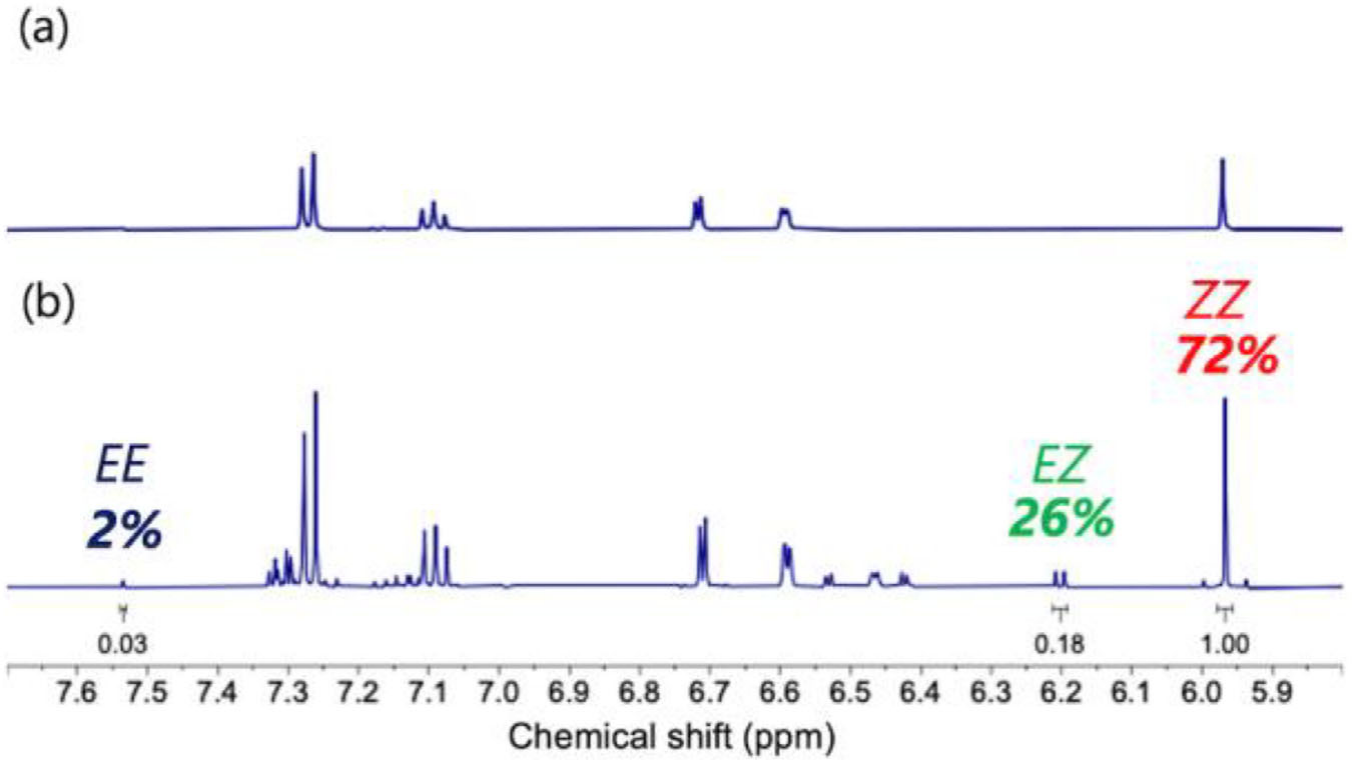
^1^H NMR (500 MHz, [*D*_12_]cyclohexane, 25°C) spectra of (a) isolated *ZZ*‐**1b** and (b) a mixture of geometrical isomers of **1b** obtained after reaching thermal equilibrium at 65°C.

Compound **1b** underwent negligible thermal isomerization at room temperature, similar to **1a**. To reach thermodynamic equilibrium, a [*D*_12_]cyclohexane solution was heated at 65°C for 3 days. At equilibrium, the *ZZ*‐isomer dominated, with a ratio of *ZZ*:*EZ*:*EE* ≈ 72:26:2, consistent with the trend observed for **1a** (Figure 2b).

Because complete separation of the three geometrical isomers was not feasible using the available separation techniques, the absorption spectrum of each individual isomer was determined by linear deconvolution of the mixture's spectrum (see Supporting Information for details). The molar extinction coefficient (ε) of *ZZ*‐**1b** was obtained directly from the spectrum of the pure *ZZ*‐**1b**, and the ε values of the *EZ*‐ and *EE*‐isomers were then extracted from the spectra of two mixtures of known composition, as determined by ^1^H NMR analysis.

The resulting absorption spectra of the three isomers of **1b** in cyclohexane are shown in Figure 3. The longest‐wavelength absorption bands including their vibronic fine‐structure attributed to the S_1_ state showed no significant differences between the three geometrical isomers. In contrast, pronounced differences were observed for the second absorption band at approximately 310 nm attributed to the S_2_ state. In this region, the molar absorption coefficients of *ZZ*‐**1b** and *EE*‐**1b** were significantly higher than those of *EZ*‐**1b**. This differential absorption indicates that irradiation of the second absorption band preferentially excites the *ZZ* and *EE*‐isomers, whereas the *EZ*‐isomer absorbs substantially less in this wavelength region.

**FIGURE 3:**
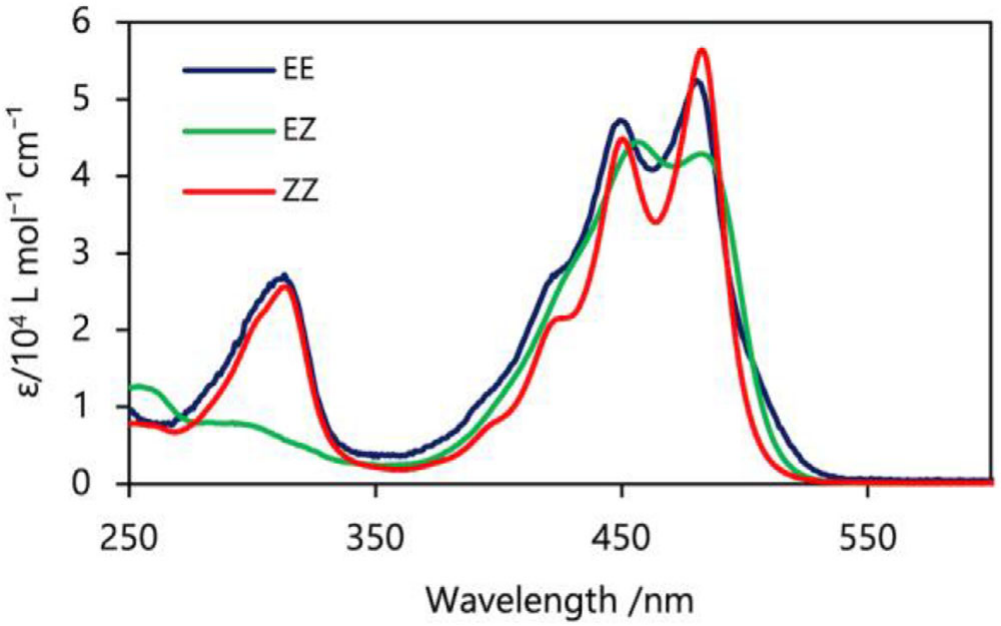
UV–vis absorption spectra of all three individual geometrical isomers (*EE*, *EZ*, and *ZZ*‐**1b**) in cyclohexane.

Comparable measurements in acetonitrile (Figure S18) reveal no significant differences in the first absorption band. However, the second absorption band of the *EE*‐isomer is noticeably broadened relative to that in cyclohexane.

Photoisomerization of **1b** was confirmed by UV‐vis absorption spectroscopy. Upon irradiation of a cyclohexane solution of pure *ZZ*‐**1b** at 312 nm, where the *EZ*‐isomer absorbed significantly less light than the other two isomers (see above), spectral changes, especially a decrease in the second absorption band around 310 nm, were observed within 3 s of irradiation, and the system reached a photostationary state (PSS) within 24 s (Figure 4). This rapid response demonstrated that **1b** underwent efficient photoisomerization upon excitation of the second absorption band.

**FIGURE 4:**
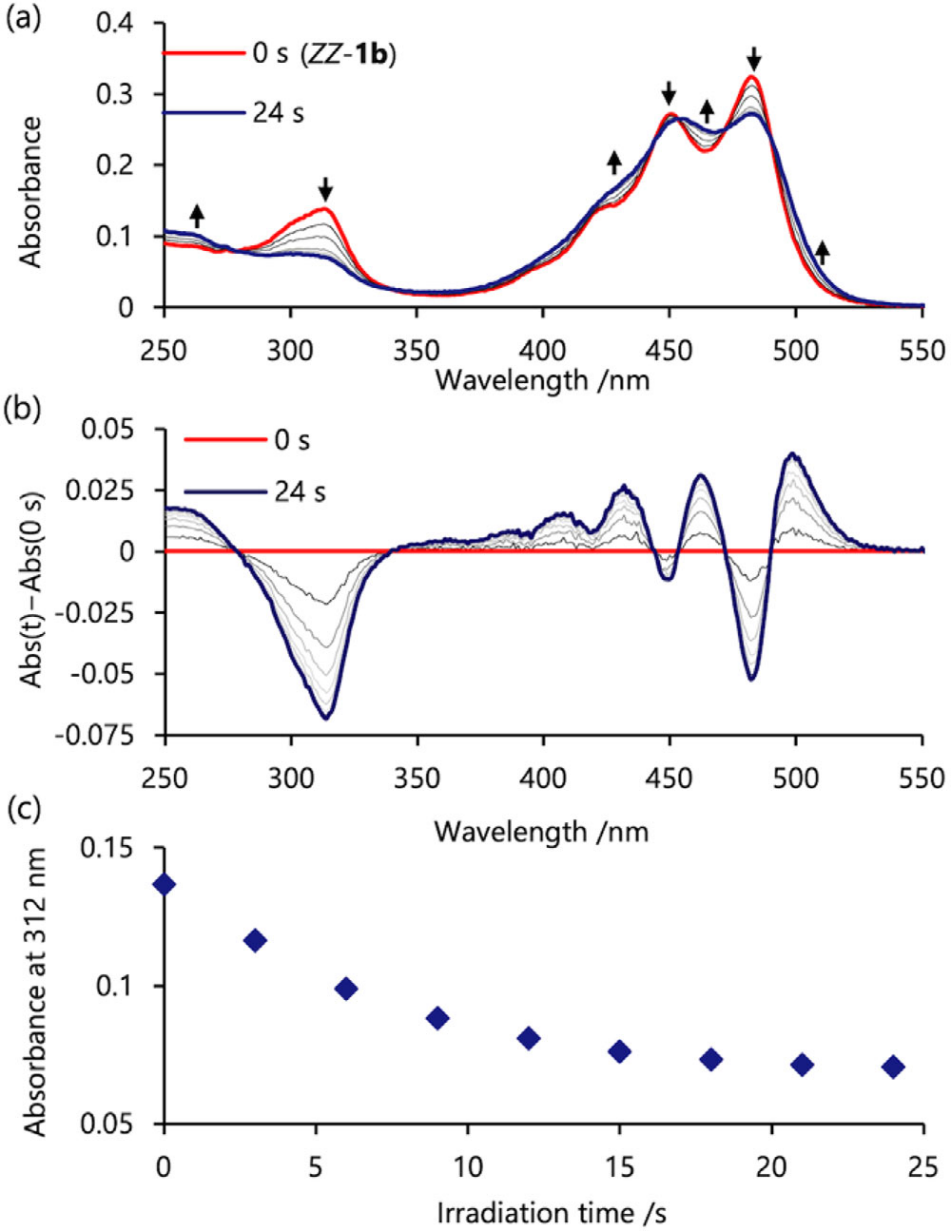
UV–vis absorption spectral changes upon irradiation of *ZZ*‐**1b** in cyclohexane at 25°C with 312 nm light. (a) Absorption spectra recorded at 3 s intervals over a total irradiation period of 24 s. (b) Difference spectra obtained by subtracting the spectrum at 0 s from each subsequent spectrum. (c) Time course of the absorbance at 312 nm.

The geometrical isomer distribution at the PSS was determined by ^1^H NMR spectroscopy. The PSS ratio upon excitation with a 310 nm LED was *ZZ*:*EZ*:*EE* ≈ 13:75:12 (Table 1). This distribution differs markedly from that at thermodynamic equilibrium (*ZZ*:*EZ*:*EE* ≈ 72:26:2), with the *EZ*‐isomer increasing from a minor component (26%) to the predominant species (75%). The pronounced accumulation of the *EZ*‐isomer in the PSS is consistent with its lower ε value of the second absorption band.

**TABLE 1:** Molar absorption coefficients (*ε* / M^−1^ cm^−1^) of each geometrical isomer at the indicated wavelength,^a^ photostationary state (PSS) compositions,^b^ and fluorescence quantum yields (*Φ*_fl_)^c^ upon irradiation at the same wavelength in cyclohexane.

*λ* (nm)	*ε* (10^4^ M^−1^ cm^−1^)			PSS compositions (%)			*Φ*_fl_ (PSS)
	*ZZ*	*EZ*	*EE*	*ZZ*	*EZ*	*EE*	
530	0.04	0.05	0.16	47	47	6	—^d^
470	3.80	4.13	4.38	28	58	15	0.31
405	0.96	1.31	1.54	32	56	12	0.33
310	2.48	0.63	2.66	13	75	12	0.30
265	0.70	1.06	0.78	33	55	12	—^d^

^a^ Determined by linear deconvolution of mixture spectra in cyclohexane.

^b^ Determined by ^1^H NMR in CDCl_3_ after irradiation in cyclohexane with LEDs.

^c^ Determined by an absolute method using an integrating sphere; values correspond to the PSS mixture at each excitation wavelength.

^d^ Not measured.

Repeated ^1^H NMR measurements during the determination of the isomer distribution revealed no detectable decomposition even after prolonged irradiation for 17 min, indicating that **1b** possesses sufficient photostability to function as a photoswitch (Figure S15).

In acetonitrile, photoisomerization proceeded similarly, although nearly 20 min was required to reach the PSS (Figure S19), indicating slower photoisomerization kinetics as compared to the one in cyclohexane. The isomer ratio at the PSS in acetonitrile was *ZZ*:*EZ*:*EE* ≈ 9:71:19 (Figure S14). The proportion of the *EE*‐isomer was higher than in cyclohexane, reflecting the broadened absorption profile of the *EE*‐isomer in the second band in this solvent.

To explore the scope of excitation wavelength control of the photoisomerization process, the geometrical isomer ratios at the PSS in cyclohexane were determined upon irradiation at five different wavelengths (Table 1). The isomer distribution varied systematically with the excitation wavelength. Irradiation at 310 nm resulted in the highest enrichment of the *EZ*‐isomer (75%). At 470 nm, the *EE*‐isomer reached its highest proportion (15%), whereas irradiation with a 530 nm LED gave a reduced proportion of the *EE*‐isomer (6%) with equal proportions of the *ZZ*‐ and *EZ*‐isomers (47%). In principle, the PSS composition is governed by both the molar absorption coefficients and the isomerization quantum yields of the isomers at each excitation wavelength. Assuming, in accordance with the Kasha–Vavilov rule, that the isomerization quantum yields are largely independent of the excitation wavelength, these variations can be attributed primarily to the different molar absorption coefficients of the three isomers, demonstrating that the PSS composition, and hence the functional state of the system, can be addressed by choosing the proper irradiation wavelength. This effect was most pronounced in the second absorption band, where the molar absorption coefficients of the *ZZ*‐ and *EE*‐isomers substantially exceeded those of the *EZ*‐isomer, so that the absorption coefficients dominate the PSS composition in this region, leading to the highest enrichment of *EZ*‐**1b** (75%) upon 310 nm irradiation. This wavelength‐selective multi‐state control provided access to isomer distributions that were unattainable under thermodynamic equilibrium conditions.

The reversibility of the switching process was then examined. Because thermal back‐isomerization of **1b** was extremely slow at room temperature [34], the photogenerated isomer distributions were essentially retained in the dark over several days. Heating a [*D*_12_]cyclohexane solution of the PSS(310 nm) mixture at 65°C drove the system back to the thermal equilibrium distribution over the course of 3 days (Figure S16), with no decomposition products detected throughout this prolonged thermal treatment, confirming that the photogenerated state cleanly relaxes to the initial isomer distribution. This high thermal persistence of each photogenerated state is a direct consequence of our molecular design and is advantageous for retaining a selected functional state. Given this slow thermal pathway, bidirectional switching was instead achieved photochemically: alternating irradiation at 310 and 405 nm interconverted the isomer distributions between the corresponding photostationary states. This photochemical cycle could be repeated at least 9 times with no detectable degradation of the sample, as monitored by UV–vis absorption spectroscopy (Figure S20), demonstrating the high fatigue resistance required for a practical photoswitch.

The fluorescence properties of **1b** were examined using isolated *ZZ*‐**1b**. In cyclohexane, **1b** exhibited bright emission with a maximum at 560 nm (Figure 5), corresponding to a Stokes shift of 2767 cm^−1^. This shift is notably smaller than that of the parent compound **1a** (3972 cm^−1^), indicating that the structural relaxation in the excited state is significantly suppressed in **1b** (Figure S21). Consistent with this reduced relaxation, the fluorescence quantum yield of the *ZZ*‐isomer was determined to be *Φ*_fl_ = 0.40 in cyclohexane, representing a substantial enhancement over the parent compound **1a** (*Φ*_fl_ < 0.01) (Table S2). This result is consistent with the suppression of nonradiative decay through restriction of the large‐amplitude torsional motion of the aryl groups, supporting our initial hypothesis. In marked contrast, the fluorescence was almost entirely quenched in acetonitrile. Across a series of solvents, the fluorescence quantum yield decreased monotonically with increasing solvent polarity (*Φ*_fl_ = 0.40, 0.10, 0.011, and 0.005 in cyclohexane, toluene, dichloromethane, and acetonitrile, respectively; Table S2 and Figure S25a), with a growing contribution on the longer‐wavelength side of the emission band in more polar solvents (Figure S25b), indicating that a nonradiative deactivation pathway becomes increasingly dominant in polar media. The observed behavior suggests the involvement of a polar dark state with intramolecular charge‐transfer character. Notably, the enhanced quenching in polar media was accompanied by less efficient photoisomerization (Figure S19), indicating that the fluorescence is quenched not by photoisomerization but by a nonproductive nonradiative decay channel that is distinct from the reaction coordinate leading to isomerization. A detailed investigation of this solvent‐dependent quenching mechanism will be reported elsewhere.

**FIGURE 5:**
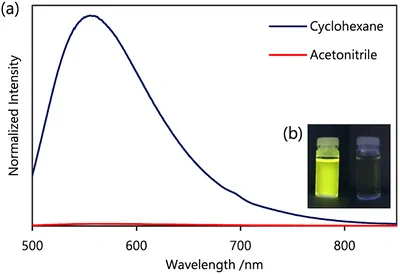
(a) Emission spectra of *ZZ*‐**1b** in cyclohexane (blue, *λ*_ex_ = 450 nm) and acetonitrile (red, *λ*_ex_ = 489 nm). The spectra are corrected for absorbance at the excitation wavelength so that the relative area underneath the curves reflects the ratio of the fluorescence quantum yields. (b) Photographs of the corresponding solutions under UV irradiation.

During the absolute quantum yield measurements under continuous excitation, the quantum yield changed gradually, eventually converging to a constant value. This behavior is attributed to photoisomerization occurring during the measurement. As the initially pure *ZZ* sample evolves toward the PSS composition, the measured quantum yield reflects a weighted average of the emission efficiencies of the individual isomers. The converged *Φ*_fl_ values at the PSS are shown in Table 1, and those values (0.30–0.33) are consistently lower than the value for pure *ZZ*‐**1b** (0.40), indicating that the *EZ*‐ and *EE*‐isomers possess lower fluorescence quantum yields. The observed trend across the three excitation wavelengths indicates that the quantum yields decreased in the order *ZZ* > *EZ*, *EE*. Fluorescence lifetime measurements further support our hypothesis of isomer‐dependent excited‐state dynamics. The fluorescence decay of *ZZ*‐**1b** in cyclohexane was fitted with a biexponential function (*τ* = 0.23 and 2.65 ns), while the decay of the irradiated sample at the PSS required a triexponential fit, with an additional longer‐lived component (*τ* = 3.45 ns) that is absent in the pure *ZZ*‐isomer (Table S3). These results indicate that the geometrical isomers indeed possess distinct excited‐state lifetimes.

## 3 Conclusion

The present study demonstrates that a thienoquinoid scaffold is able to provide both, multi‐site photoisomerization and significant fluorescence—two features that are notoriously difficult to unite within one and the same π‐conjugated molecule. The introduction of 2,6‐dichlorophenyl groups led to a pronounced enhancement in the fluorescence quantum yield and a substantial reduction in the Stokes shift, while preserving the multi‐photoisomerizable character of the scaffold. The key finding is that the photostationary state composition varies systematically with the irradiation wavelength, primarily reflecting the wavelength‐dependent differences in the absorption profiles of the three geometrical isomers, most markedly in the second absorption resembling the S_2_ state, where the selective enrichment of the *EZ*‐isomer is maximized. This wavelength‐addressable control provided access to isomer distributions that were unattainable at thermal equilibrium, thereby effectively expanding the accessible state space of the system. Fluorescence lifetime measurements further revealed isomer‐dependent excited‐state dynamics, underscoring the intimate coupling between molecular geometry and photophysical properties within this conjugated framework. The pronounced solvent‐dependent fluorescence quenching observed in acetonitrile, where both emission and photoisomerization are suppressed, indicates a distinct nonradiative pathway that becomes dominant in polar media and that may aid the design of environmentally responsive luminescent materials. The extension of our thienoquinoid‐based design strategy to longer oligomeric or even polymeric architectures with an increased number of photoaddressable states is the next step toward multi‐level optical control systems.

## Conflicts of Interest

The authors declare no conflicts of interest.

## Supporting information



The authors have cited additional references within the Supporting Information [35, 36, 37, 38].
**Supporting File 1**: chem71423‐sup‐0001‐SuppMat.pdf

## Data Availability

The data that support the findings of this study are available in the supplementary material of this article
