# Supplementary material for: Multi‐state Photoswitching in Thienoquinoid‐Based Fluorescent Trithiophenes
Source: Chemistry. 2026 Jul 17;32(39):e71423. doi: 10.1002/chem.71423 (PMC13715056; doi:10.1002/chem.71423)
Supplement: Supplementary file 1 [file CHEM-32-e71423-s001.pdf]

Supporting Information for

Multi-State Photoswitching in Thienoquinoid-Based Fluorescent Trithiophenes

Rio Nishimura,[a] Stefan Hecht,[b,c] Shohei Saito,[a] Ken-ichi Yamashita*[a,d]

[a] Department of Chemistry, Graduate School of Science, The University of Osaka, 1-1 Machikaneyama, Toyonaka, Osaka, 560-0043, Japan

[b] Department of Chemistry, Humboldt-Universität zu Berlin, Brook-Taylor-Str. 2, 12489 Berlin, Germany

[c] Center for the Science of Materials Berlin, Humboldt-Universität zu Berlin, Zum Großen Windkanal 2, 12489 Berlin, Germany

[d] Innovative Catalysis Science Division, Institute for Open and Transdisciplinary Research Initiatives (ICS-OTRI), The University of Osaka, Suita, Osaka 565-0871, Japan

E-mail: yamashita-k@chem.sci.osaka-u.ac.jp

Contents

1. Experimental details 2

2. Additional spectroscopic data 6

3. X-Ray crystal structure determinations 20

4. Theoretical calculations 23

1. Experimental details

Instrumentation and Materials

Unless otherwise noted, all the reactions were performed in a nitrogen atmosphere using anhydrous solvents and heat-gun-dried glassware on a dual-manifold Schlenk line. All other chemicals were of reagent grade and were used without any further purification unless otherwise noted. Compound **1a** was synthesized according to previously reported procedures.[32] Thin-layer chromatography (TLC) was performed on silica gel 60 F_{254} plates (Merck). Column chromatography was performed using silica gel 60N (Kanto Chemical, spherical, neutral, 63–210 µm). All NMR spectral data were recorded on a JEOL ECA-500 (500 MHz for ^{1}H, 125 MHz for ^{13}C), JEOL ECA-400 (400 MHz for ^{1}H) or a Bruker DPX 400 (400 MHz for ^{1}H) spectrometers at ambient temperature (25 °C). ^{1}H NMR spectra were referenced to tetramethylsilane as a standard. ^{13}C NMR spectra were referenced internally to a solvent signal (δ = 77.0 ppm for $CDCl_3$). High-resolution electrospray ionization mass spectrometry (ESI-HRMS) data were obtained using a Thermo Fisher Scientific Q-Exactive mass spectrometer.

Photophysical measurements

UV–vis spectroscopy was performed on an Agilent Cary 60 instrument or a Shimadzu UV-3600 spectrometer using 10 mm × 10 mm quartz cuvettes (3 mL volume). For the UV light irradiation experiments conducted using the Cary 60 setup, an Oriel 500 W Hg(Xe) short-arc lamp (model LSB740) housed in an Oriel arc lamp housing (LSH302) was used. The Cary 60 system was equipped with an Oriel 300 mm grating monochromator (model MSH-300), a shutter, and a light guide. Fluorescence spectroscopy was performed on a JASCO FP-8200 spectrometer. Absolute fluorescence quantum yields were determined using a HAMAMATSU C9920-02S instrument. In particular, to minimize the influence of structural changes, the fluorescence quantum yield of *ZZ*-**1b** was determined using a relative method with fluorescein (Φ_{fl} = 0.88 in 0.10 M NaOH (aq)) as the reference standard. Fluorescence lifetimes were recorded on a HORIBA DeltaFlex time-correlated single-photon counting (TCSPC) system using a Delta Diode for excitation (405 nm).

Measurement of Isomer Ratio at Thermal Equilibrium

The equilibrium ratios of the geometrical isomers were determined as follows: NMR samples were prepared in sealed, light-shielded NMR tubes using a deuterated solvent ([D_{12}]cyclohexane). The samples were heated in a metal bath at a designated temperature outside the NMR instrument. Thermal equilibrium was confirmed by performing multiple measurements over time until no further changes in the isomer ratios were observed. Prior to NMR analysis, the heated samples were rapidly cooled to room temperature (r.t.) and promptly measured at 25 °C.

This rapid cooling approach is valid because, as demonstrated in our previous study,[32] the interconversion between the isomers at r.t. is negligibly slow, with no significant changes observed over several days. Therefore, the "frozen" equilibrium state accurately reflects the isomer distribution at the elevated temperature.

Measurement of Isomer Ratio at Photostationary State (PSS)

The geometrical isomer ratios in the photostationary state were determined as follows: *ZZ*-**1b** was dissolved in cyclohexane and irradiated using a mounted light-emitting diode (LED; Thorlabs). The irradiation wavelengths and the specifications of the LEDs used are summarized in Table S1. After irradiation, the solvent was rapidly removed under light-shielded conditions and reduced pressure, and ^{1}H NMR spectra were recorded in $CDCl_3$ at r.t.. Irradiation was continued until no further changes in the isomer ratios were observed, and the photostationary state was confirmed by performing multiple ^{1}H NMR measurements.

Table S1. LED specifications

**Wavelength**	**Item**	**Peak Wavelength / nm**	**Maximum Irradiance / µW mm^{-2}**	**Bandwidth (FWHM) / nm**
265 nm	M265L5	265	0.5	11
310 nm	M310L1	308	0.76	30
405 nm	M405L4	405	24.6	12.5
450 nm	M450LP2	450	34.2	18
470 nm	M470L5	470	21.4	28
530 nm	M530L4	530	9.46	35

Determination of the Absorption Spectra of Each Isomer

Because complete separation of the geometrical isomers was not achieved, the absorption spectra of each isomer were determined according to the following procedure: Measurements were performed using cyclohexane and acetonitrile as solvents. The molar extinction coefficient of the *ZZ* isomer was determined using *ZZ*-**1b**. Subsequently, the molar extinction coefficients were obtained for two samples. The geometrical isomer ratios of each sample were determined by ^{1}H NMR, and by taking these ratios into account, the molar extinction coefficients of the *EZ*-**1b** and *EE*-**1b** isomers were calculated by spectral deconvolution.

Synthetic Procedures

Synthesis of 2,5-bis((2,6-dichlorophenyl)hydroxymethyl)thiophene (2b)

To a 100 mL two-necked flask, *n*-hexane (25 mL), *N*,*N*,*N'*,*N'*-tetramethylethylenediamine (3.8 mL, 2.5 eq.), and *n*-BuLi (2.69 M solution in hexane, 9.5 mL, 2.5 eq.) were added. After stirring at r.t. for 10 min, thiophene (0.80 mL, 1 eq.) was added to the mixture, which was then stirred for another 60 min while being heated to 50°C. After cooling the reaction solution in an ice bath, a solution of 2,6-dichlorobenzaldehyde (4.5 g, 2.5 eq.) in THF (25 mL) was added dropwise, and the mixture was stirred for 180 min. The reaction was then quenched with an aqueous ammonium chloride solution. The reaction mixture was diluted with ethyl acetate, washed with brine, dried over Na_2SO_4, and concentrated under reduced pressure. Purification by column chromatography (Silica gel 60N, hexane: ethyl acetate = 4:1) yielded a brown solid (3.57 g, 81%). NMR analysis suggested that the product was a mixture of diastereomers.

^{1}H NMR (500 MHz, $CDCl_3$) δ = 7.33 (d, 4H), 7.20 (m, 2H), 6.68 (m, 2H), 6.51 (s, 2H), 3.75 ppm (s, 2H); ^{13}C NMR (125 MHz, $CDCl_3$) δ = 145.6 (*C*q), 136.8 (*C*q), 134.9 (*C*q), 129.9 (*C*H), 129.5 (*C*H), 123.9 (*C*H), 70.3 ppm (*C*H); HRMS(ESI): *m/z*: calcd for $C_{18}H_{12}Cl_4O_2SNa$: 456.9175 $[M+Na]^+$, found: 456.9173.

Synthesis of 1b

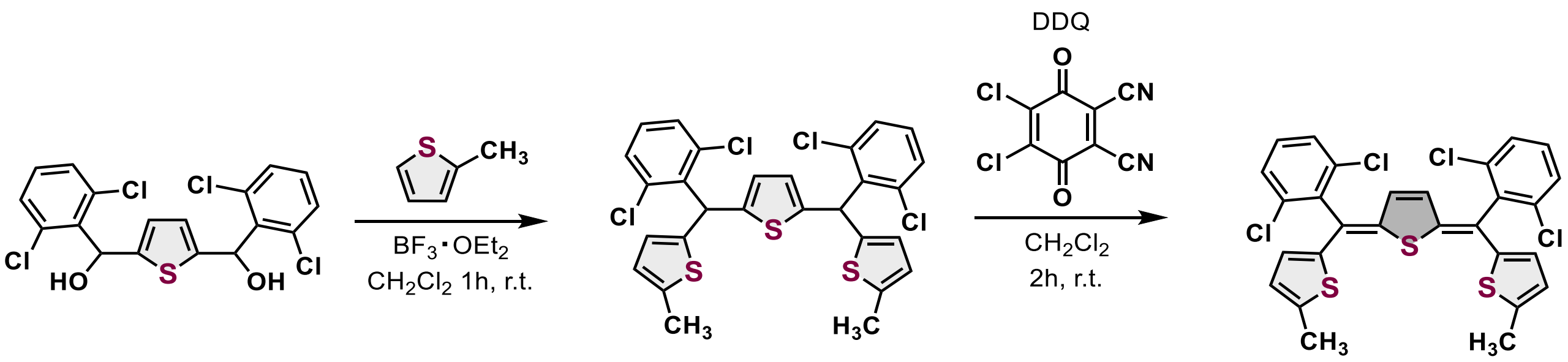

2b 1b' 1b

First step: Synthesis of **1b′**

To a 500 mL two-necked flask containing **2b** (1000.1 mg, 2.30 mmol, 1 eq), dichloromethane (230 mL), 2-methylthiophene (1.5 mL, 7.78 mmol, 3.4 eq.) and $BF_3{\cdot}OEt_2$ (145 μL, 2.31 mmol, 1.0 eq.) were added, and the mixture was stirred at r.t. for 60 min. After the reaction, the solution was washed with an aqueous $NaHCO_3$ solution, water, and brine. The organic phase was dried over Na_2SO_4, filtered, and then concentrated under reduced pressure. ^{1}H NMR analysis of the crude product confirmed that the reaction proceeded quantitatively; therefore, the product was used in the next step without further purification. NMR analysis suggested that the product was a mixture of diastereomers.

^{1}H NMR (400 MHz, $CDCl_3$) δ = 7.30 (d, 4H), 7.12 (m, 2H), 6.81 (d, 2H), 6.76 (s, 2H), 6.64 (d, 2H), 6.58 (d, 2H), 2.42 ppm (s, 6H).

Second step: Synthesis of **1b**

To a 300 mL flask, the crude product of **1b′** (1368 mg, 2.30 mmol, 1 eq.), dichloromethane (230 mL) and DDQ (574.6 mg, 2.53 mmol, 1.1 eq.) were added, and the resulting solution was stirred at r.t. for 120 min. After the reaction, purification by column chromatography (three times; silica gel 60N, hexane: chloroform = 3:1, hexane: dichloromethane = 4:1, and hexane: toluene = 2:1) afforded an orange solid **1b** as a mixture of three geometrical isomers (151.3 mg, 11%). Because the NMR spectrum of the mixture was complicated, the data are not provided here; the spectra are shown in Figure S6 and S7.

E.A. for geometrical isomeric mixture:

calcd. (%) for $C_{28}H_{18}Cl_4S_3$: C 56.77, H 3.06, N 0; found C 56.77, H 3.11, N 0.

Subsequent recrystallization of the mixture from dichloromethane/methanol mixed solvent afforded the pure *ZZ*-**1b** (81.8 mg).

ZZ-**1b**

^{1}H NMR (500 MHz, $CDCl_3$) δ = 7.41 (d, 4H), 7.28 (m, 2H), 6.75 (d, 2H), 6.73 (dd, 2H), 6.09 (s, 2H), 2.54 ppm (s, 6H); ^{13}C NMR (125 MHz, $CDCl_3$) δ (ppm) = 142.0 (*C*q), 140.4 (*C*q), 139.8 (*C*q), 137.8 (*C*q), 136.6 (*C*q), 132.3 (*C*H), 129.9 (*C*H), 128.3 (*C*H), 127.2 (*C*H), 126.4 (*C*H), 121.6 (*C*q), 15.8 ppm (*C*H_3).

2. Additional spectroscopic data

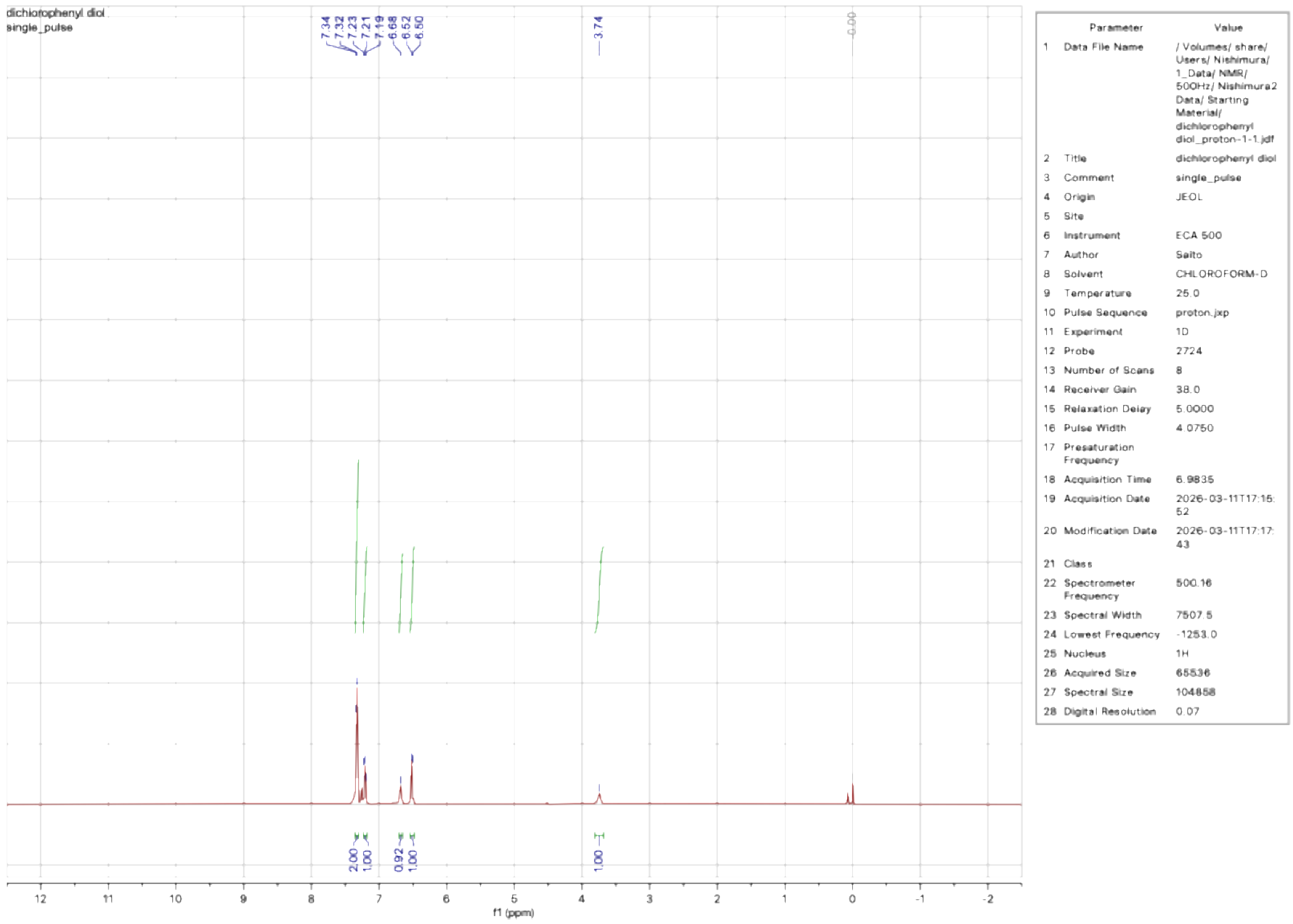

Figure S1. ^{1}H NMR spectrum (500 MHz, $CDCl_3$) of **2b**.

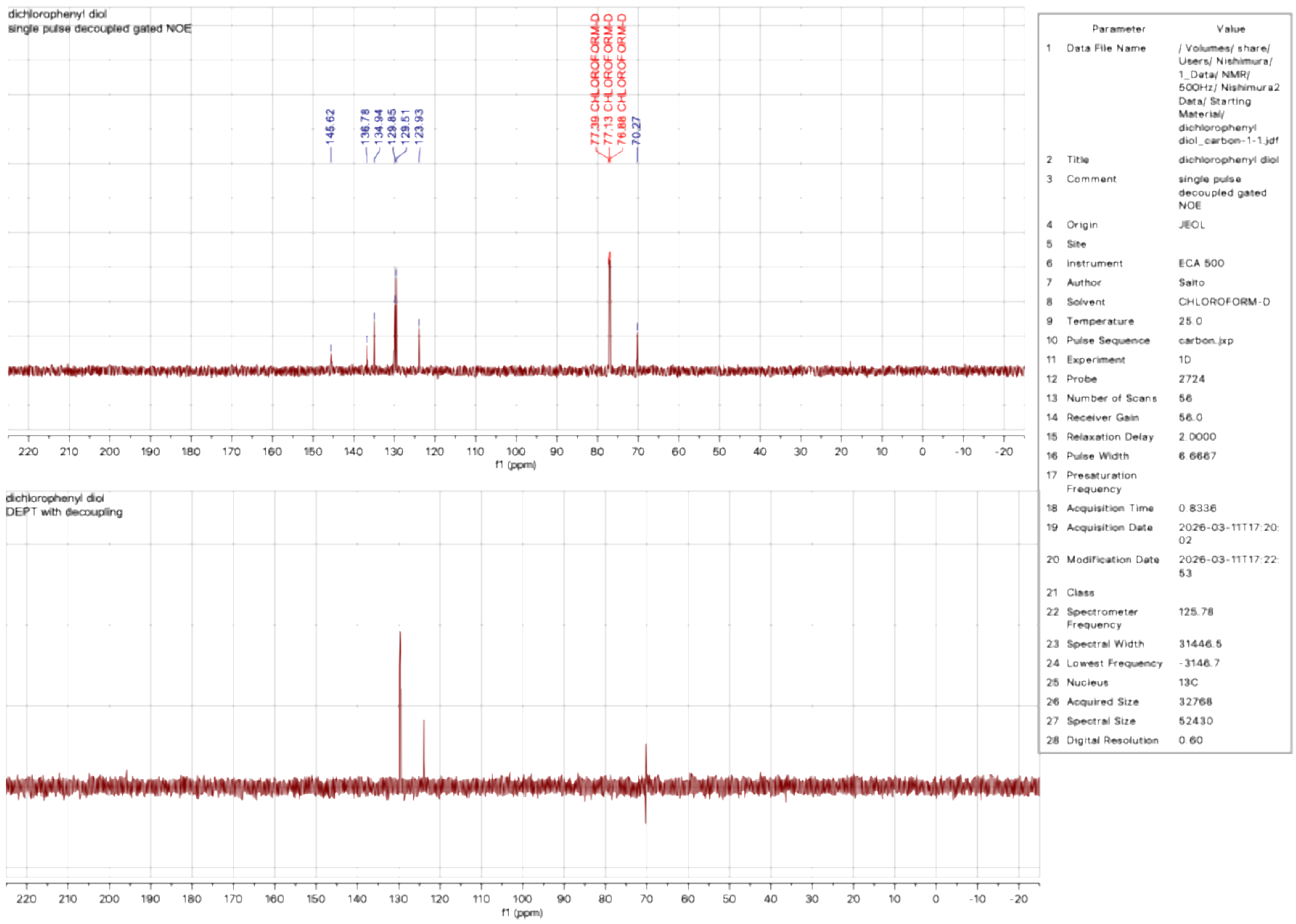

Figure S2. ^{13}C and DEPT-135 NMR spectra (125 MHz, $CDCl_3$) of **2b**.

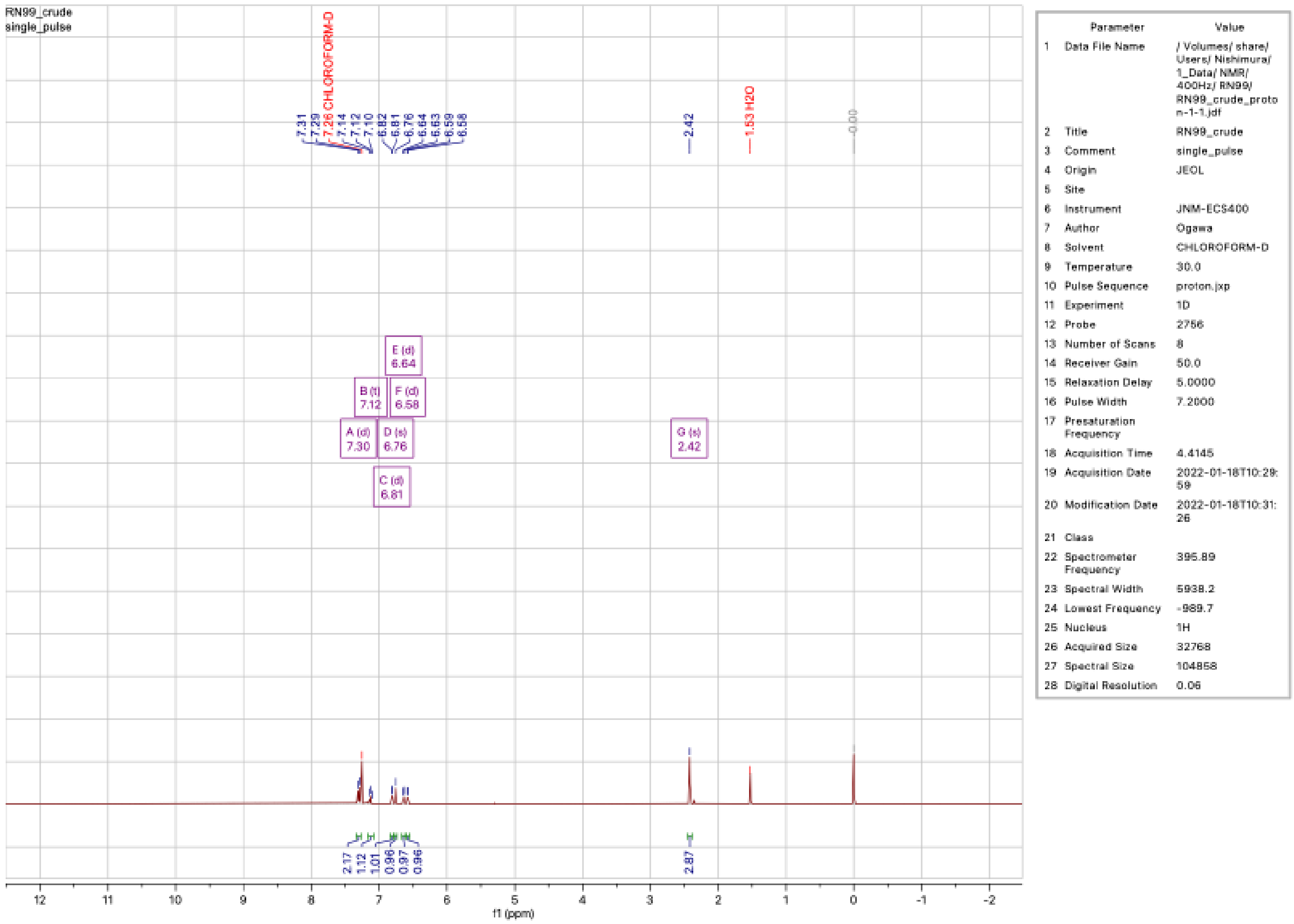

Figure S3. ^{1}H NMR spectrum (400 MHz, $CDCl_3$) of **1b′**.

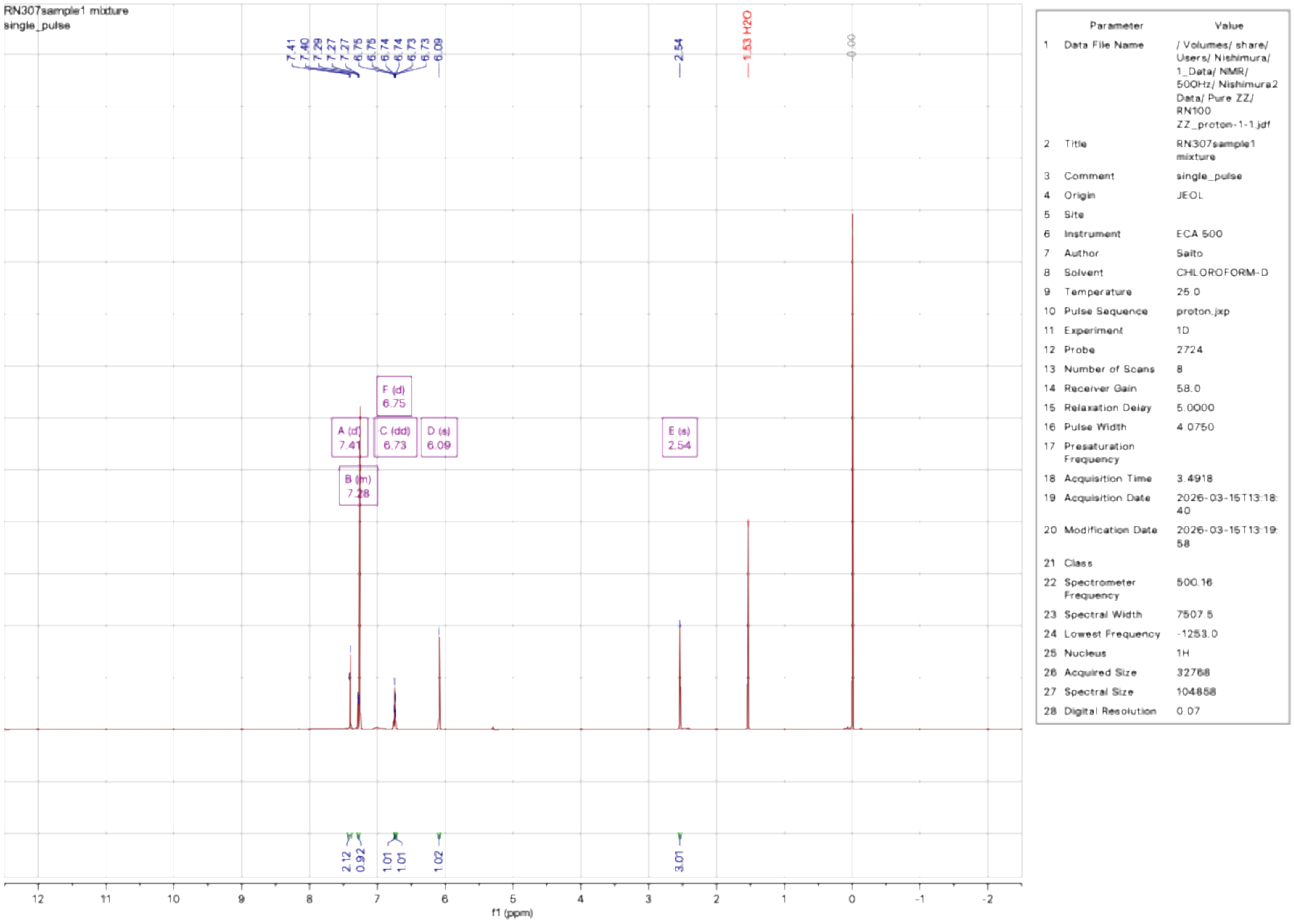

Figure S4. ^{1}H NMR spectrum (500 MHz, $CDCl_3$) of *ZZ*-**1b**.

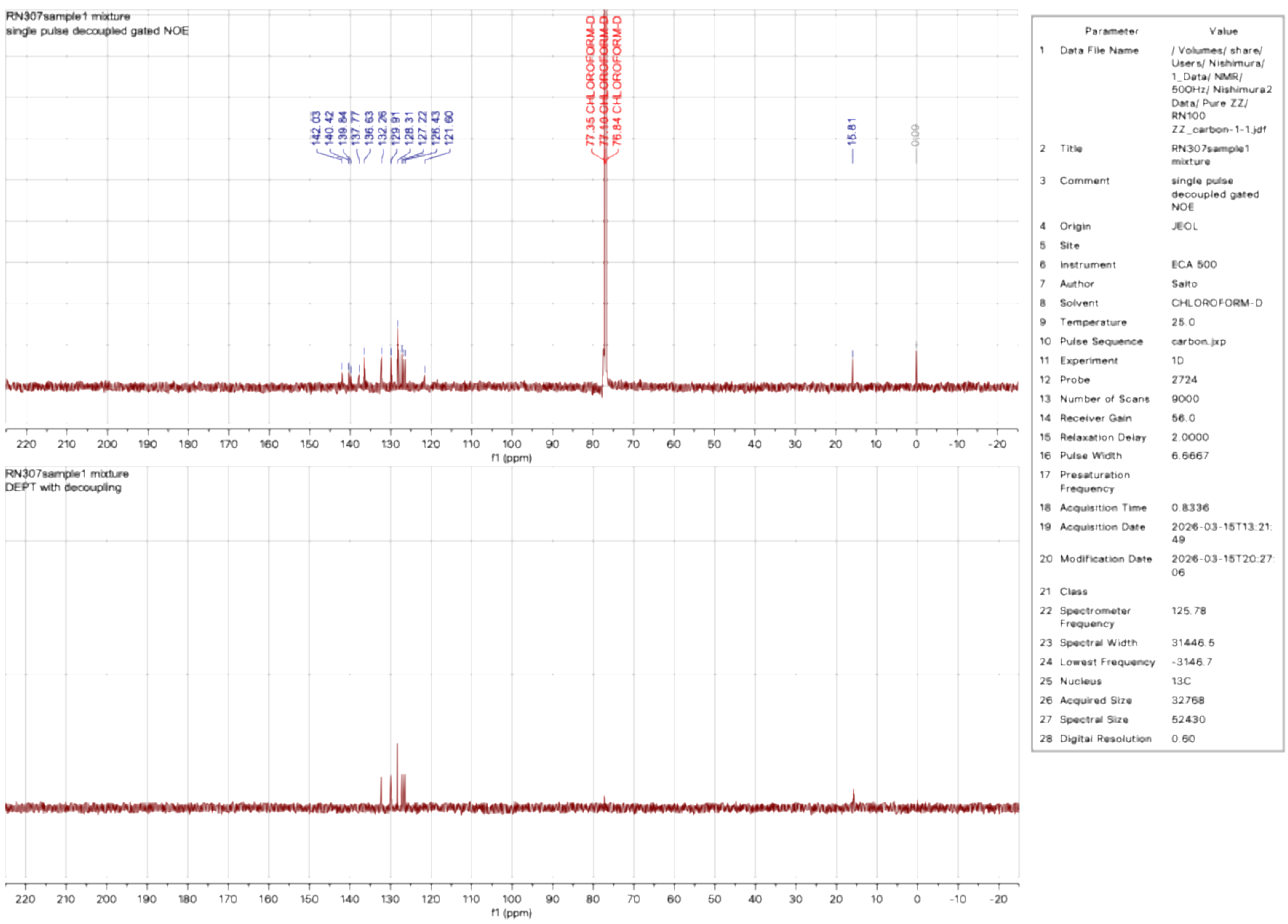

Figure S5. ^{13}C and DEPT-135 NMR spectra (125 MHz, $CDCl_3$) of *ZZ*-**1b**.

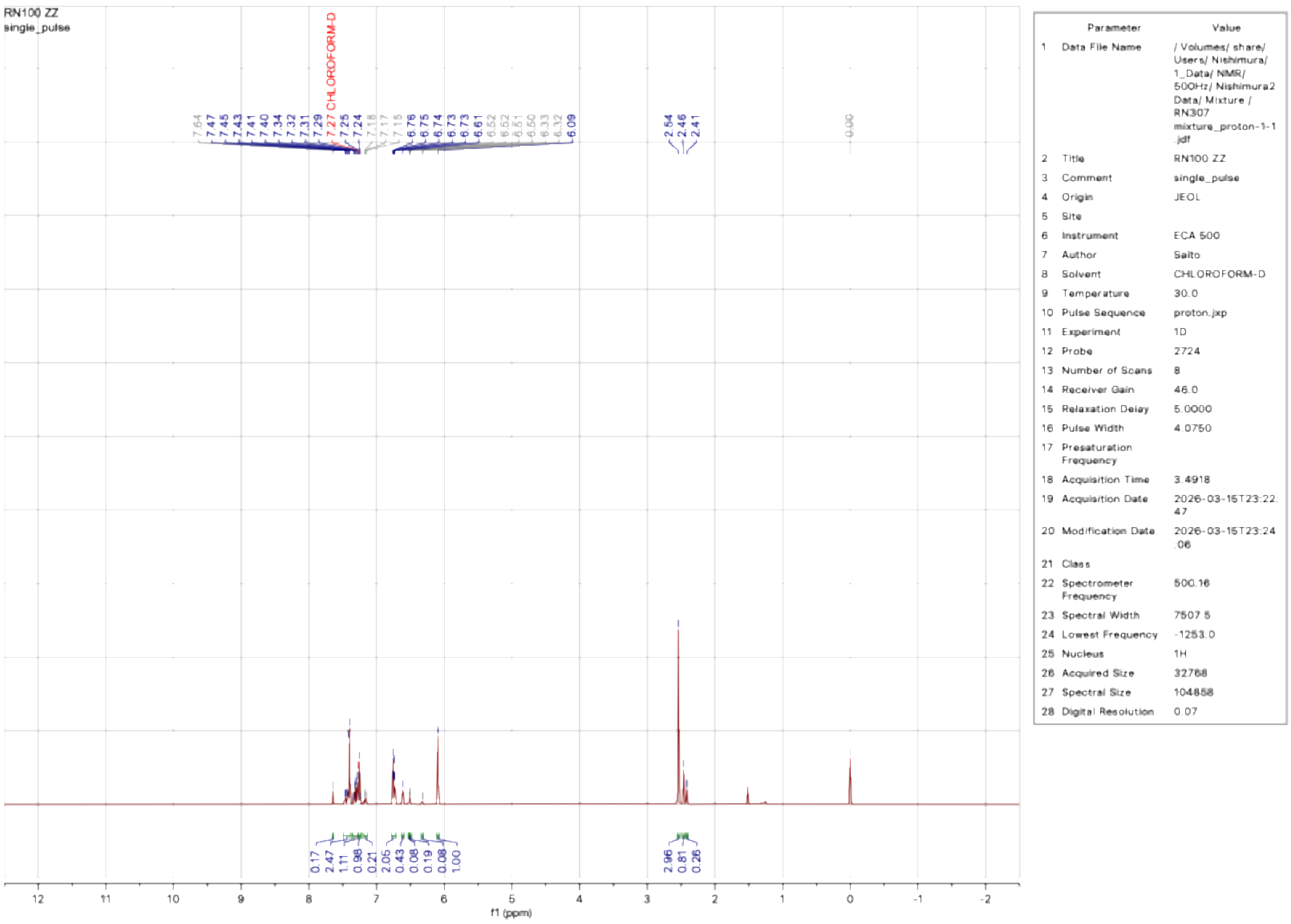

Figure S6. ^{1}H NMR spectra (500 MHz, $CDCl_3$) of the geometrical isomer mixture of **1b** (non-equilibrium state).

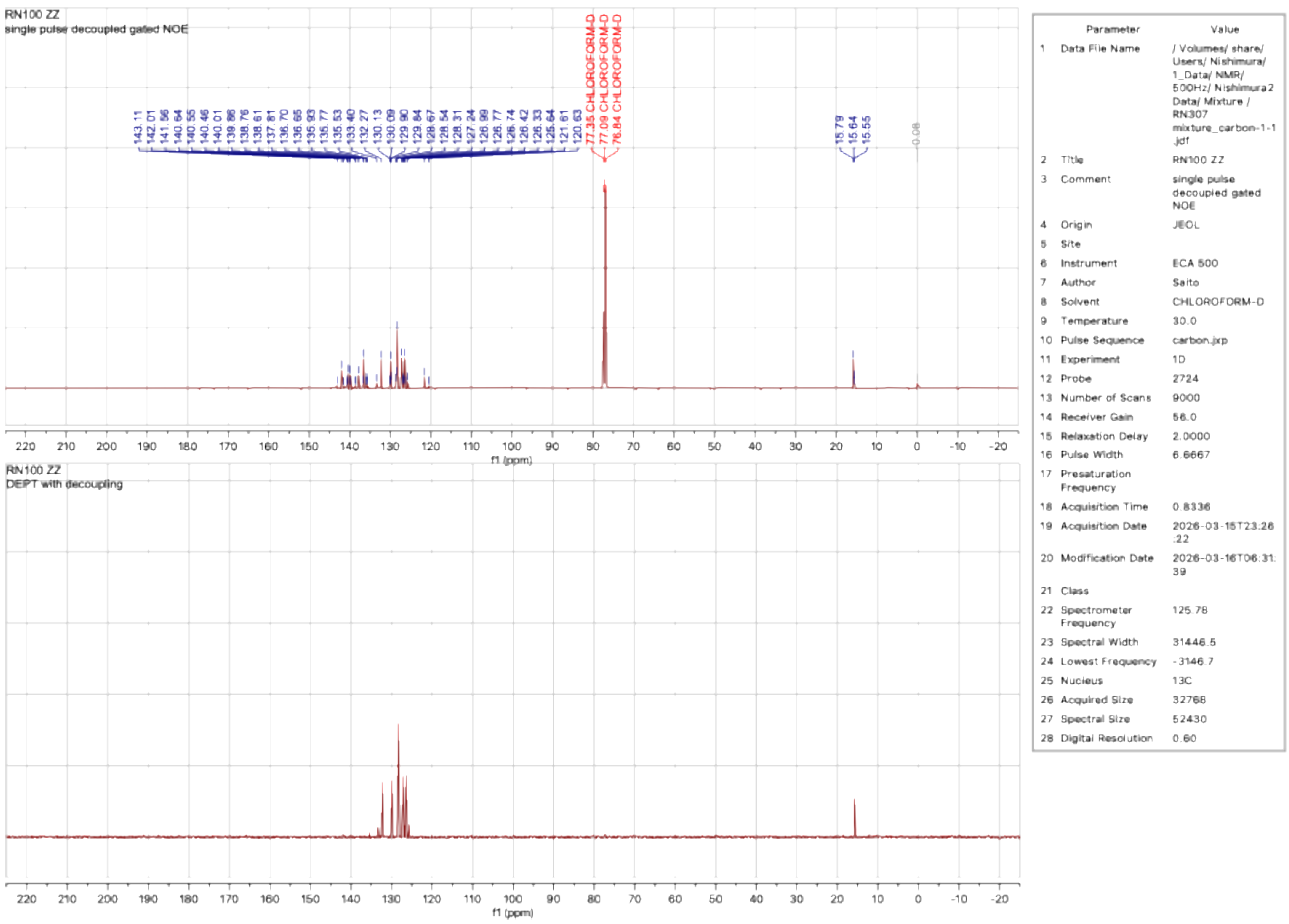

Figure S7. ^{13}C and DEPT-135 NMR spectra (125 MHz, $CDCl_3$) of the geometrical isomeric mixtures of **1b** (non-equilibrium state).

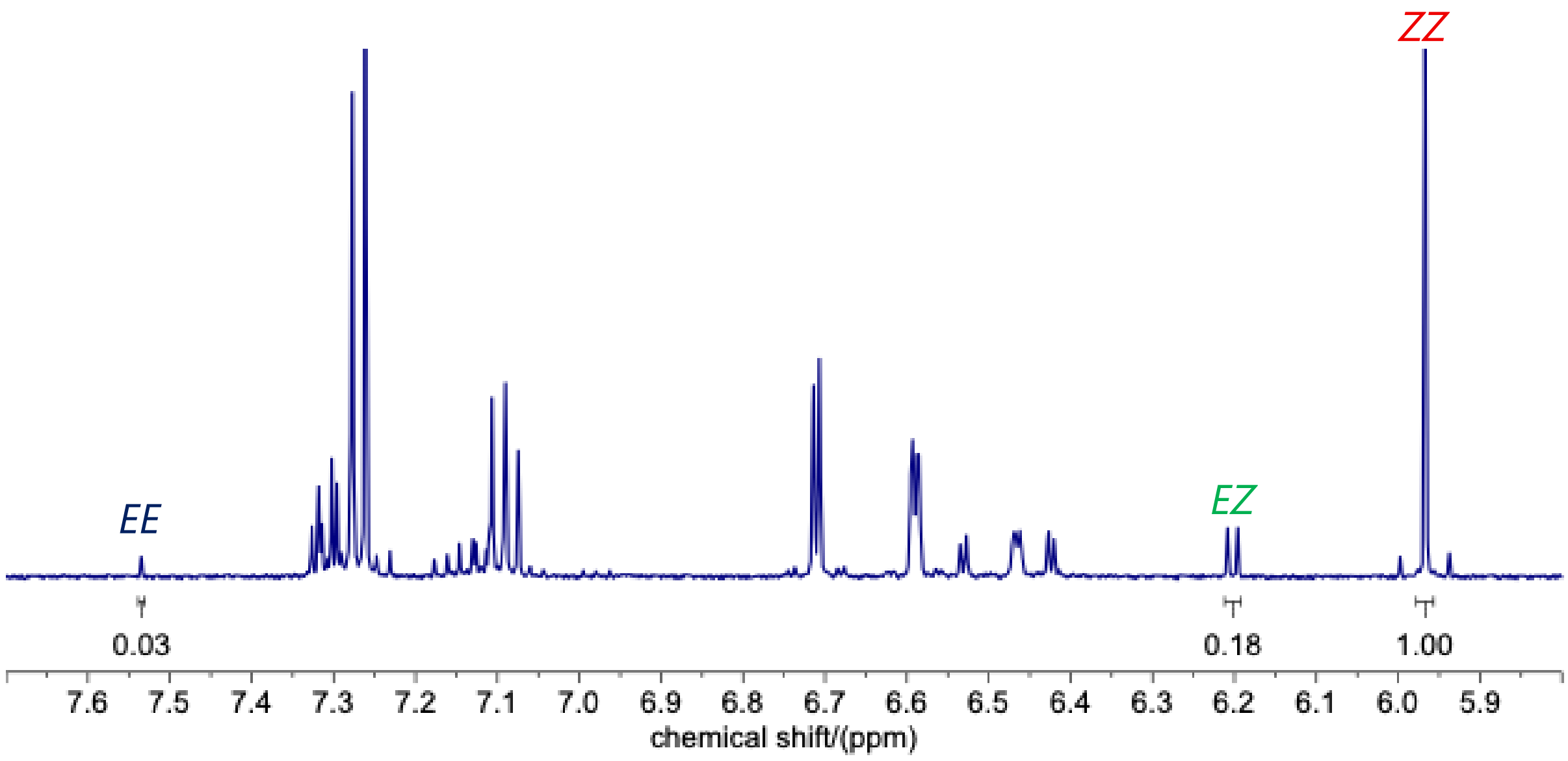

Figure S8. ^{1}H NMR spectrum (500 MHz, [D_{12}]cyclohexane) of **1b** in the thermal equilibrium state at 65°C.

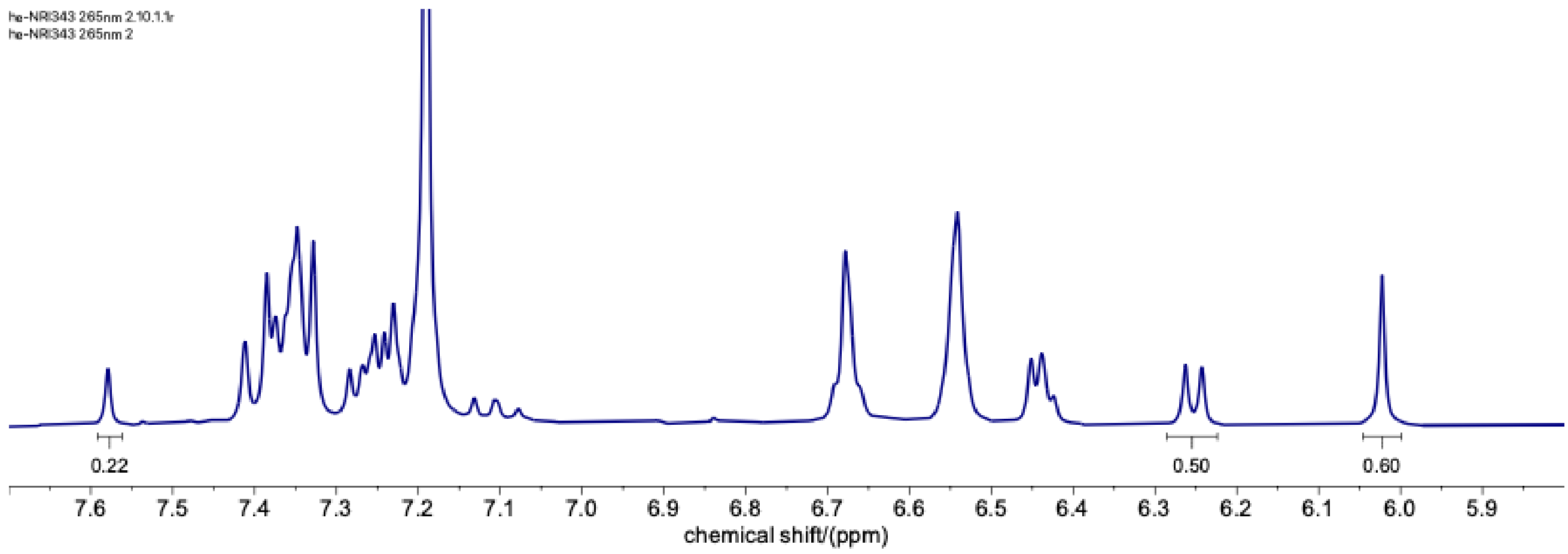

Figure S9. ^{1}H NMR spectrum (400 MHz, $CDCl_3$) of **1b** after 265 nm irradiation in cyclohexane, showing the geometrical isomer ratios at the photostationary state (PSS).

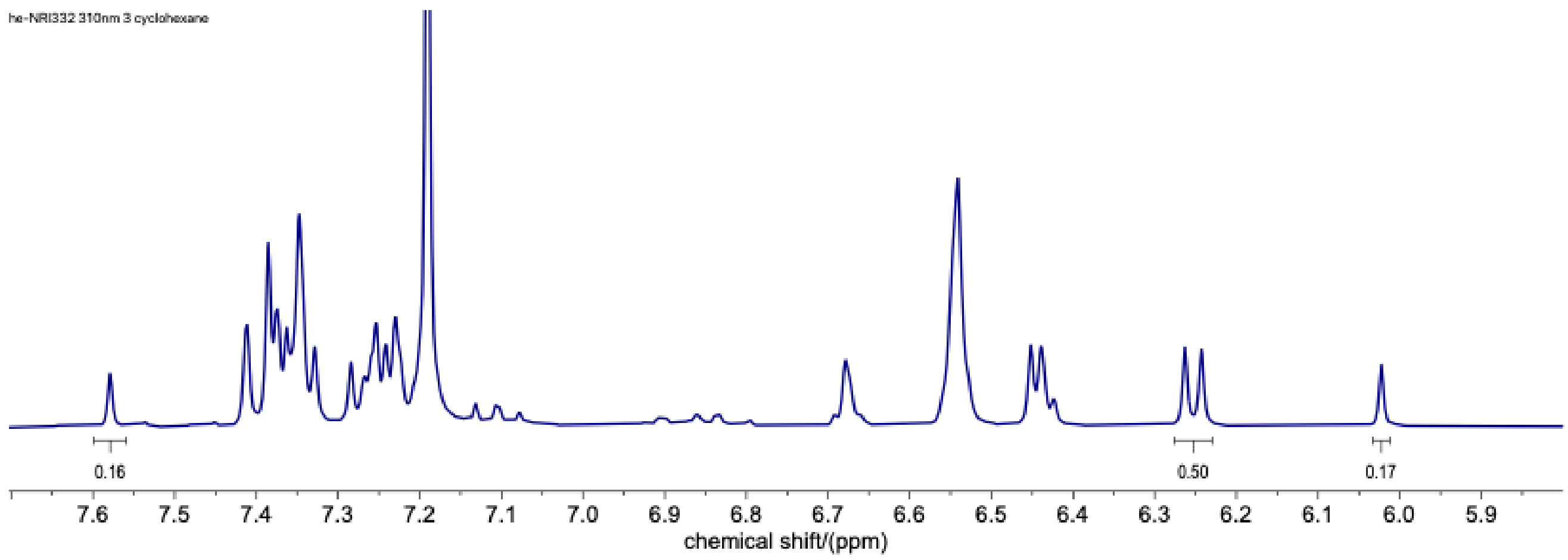

Figure S10. ^{1}H NMR spectrum (400 MHz, $CDCl_3$) of **1b** after 310 nm irradiation in cyclohexane, showing the geometrical isomer ratios at the photostationary state (PSS).

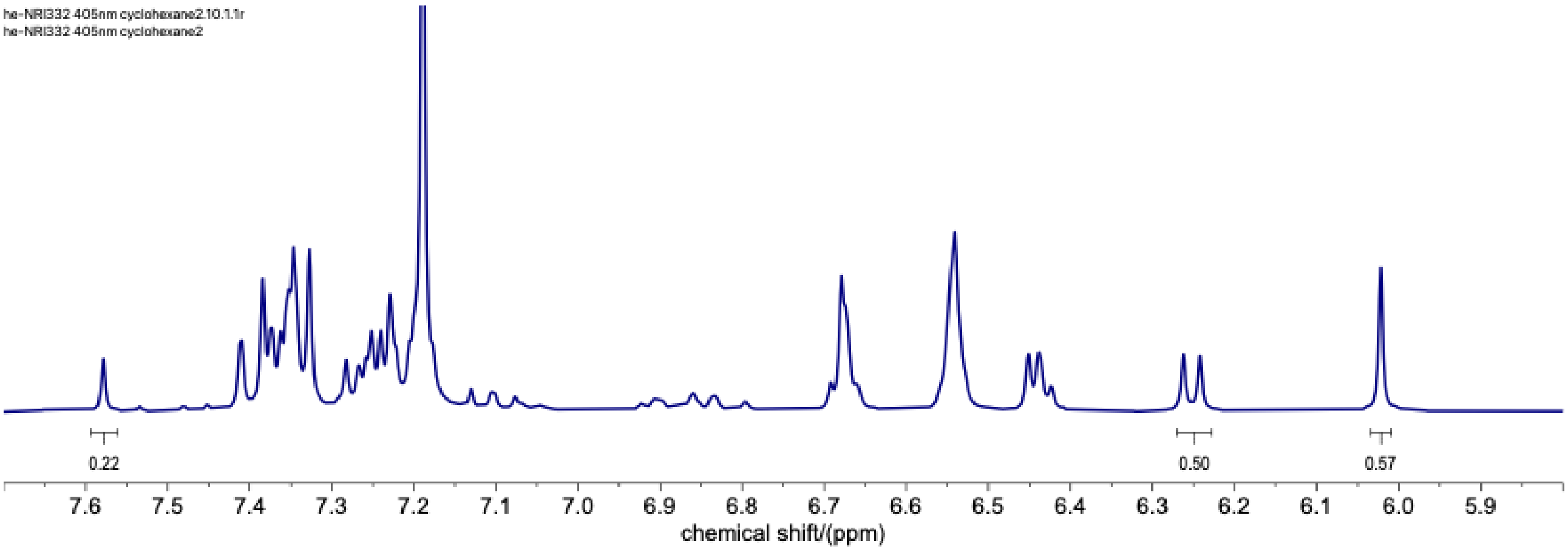

Figure S11. ^{1}H NMR spectrum (400 MHz, $CDCl_3$) of **1b** after 405 nm irradiation in cyclohexane, showing the geometrical isomer ratios at the photostationary state (PSS).

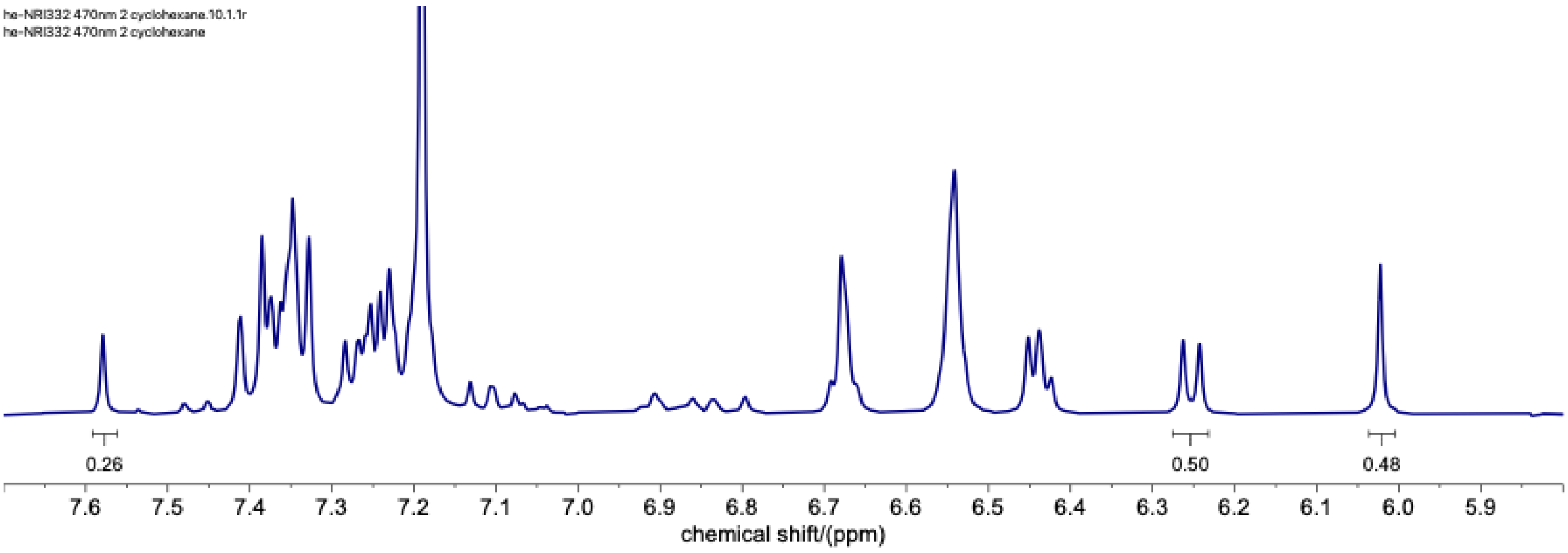

Figure S12. ^{1}H NMR spectrum (400 MHz, $CDCl_3$) of **1b** after 470 nm irradiation in cyclohexane, showing the geometrical isomer ratios at the photostationary state (PSS).

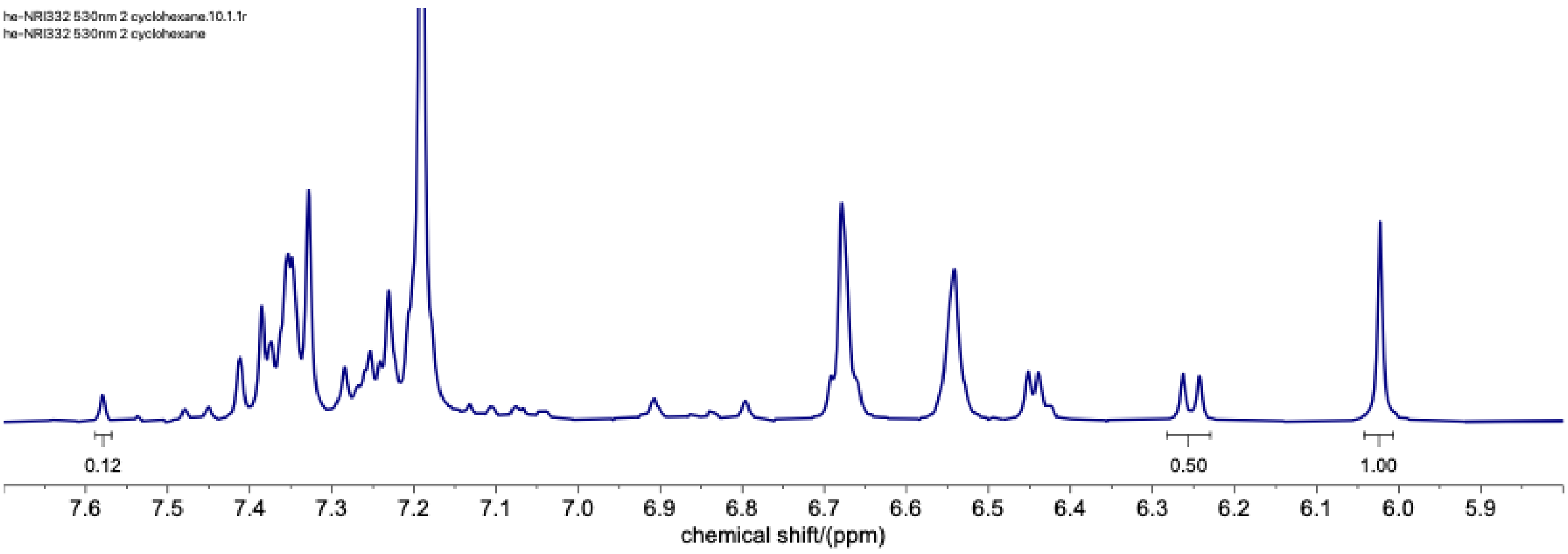

Figure S13. ^{1}H NMR spectrum (400 MHz, $CDCl_3$) of **1b** after 530 nm irradiation in cyclohexane, showing the geometrical isomer ratios at the photostationary state (PSS).

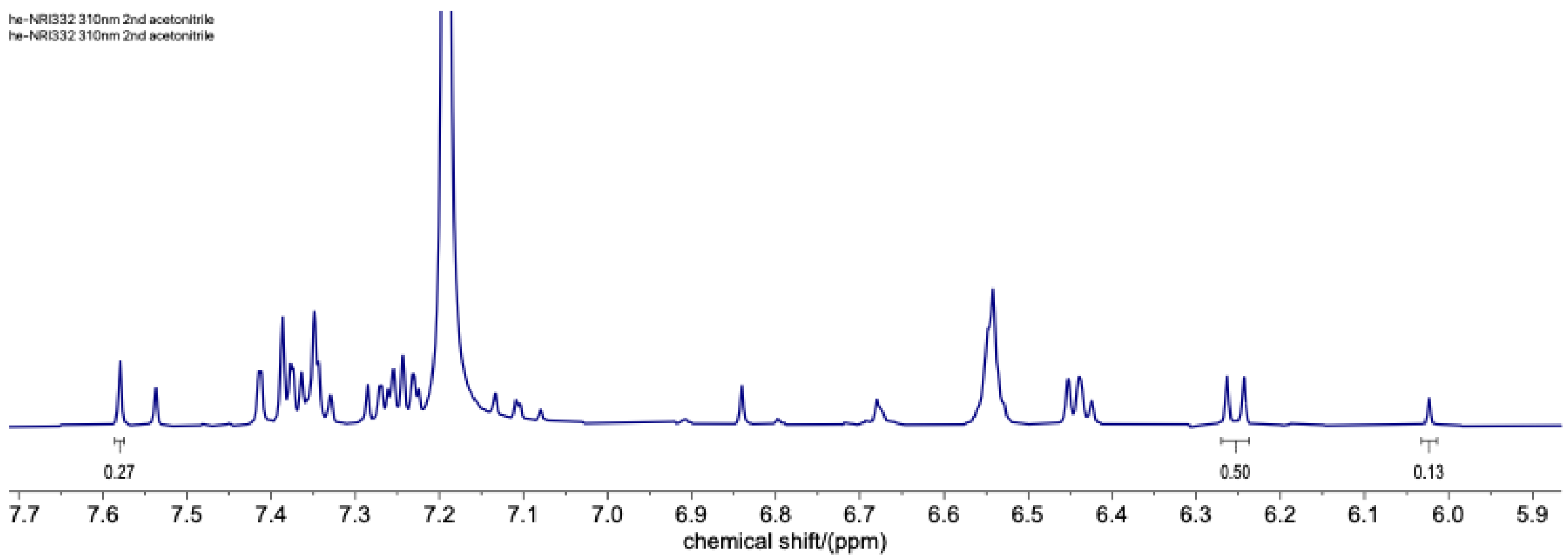

Figure S14. ^{1}H NMR spectrum (400 MHz, $CDCl_3$) of **1b** after 310 nm irradiation in acetonitrile, showing the geometrical isomer ratios at the photostationary state (PSS).

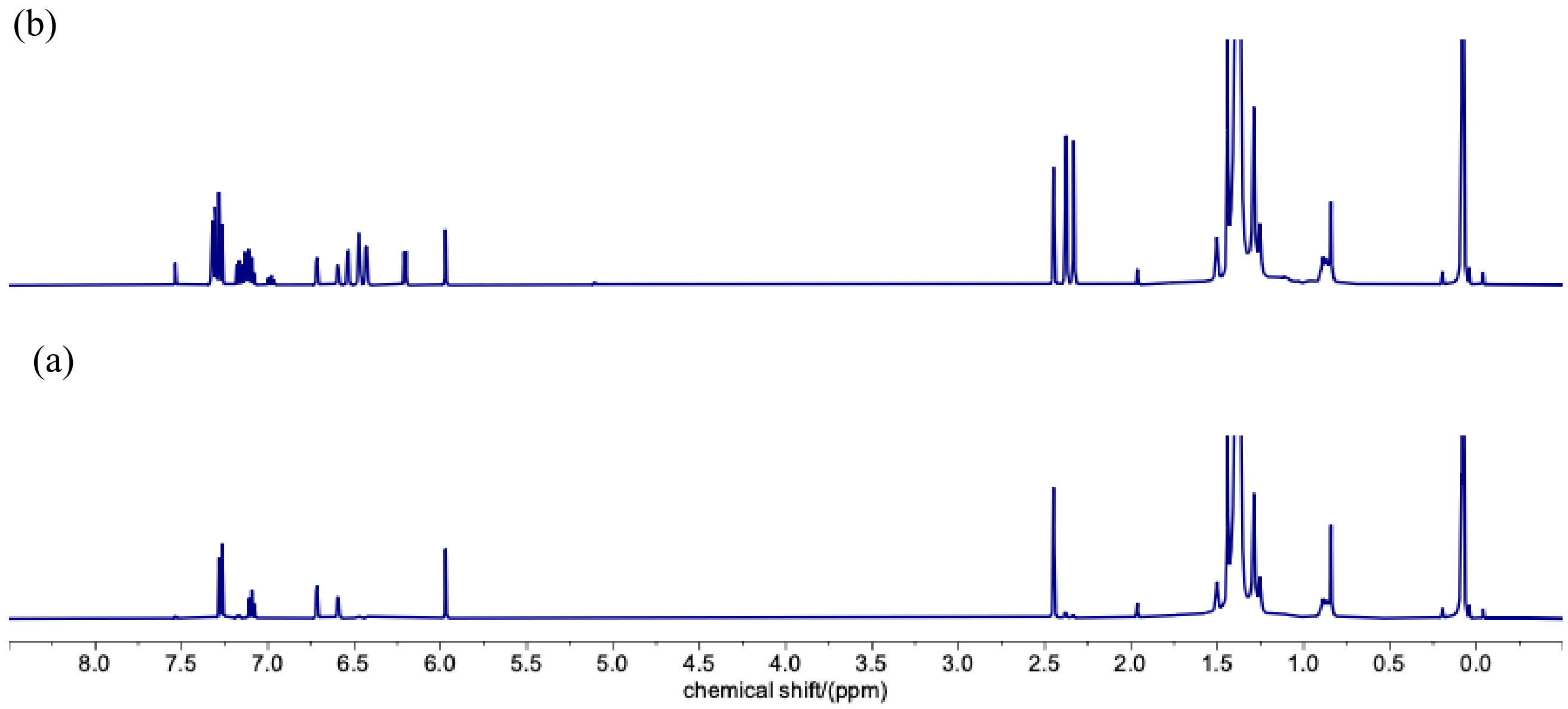

Figure S15. ^{1}H NMR spectra (500 MHz, [D_{12}]cyclohexane) of **1b** (a) before irradiation (pure *ZZ*) and (b) after irradiation at 310 nm for 17 min. No significant decomposition products were observed even after 17 min of irradiation.

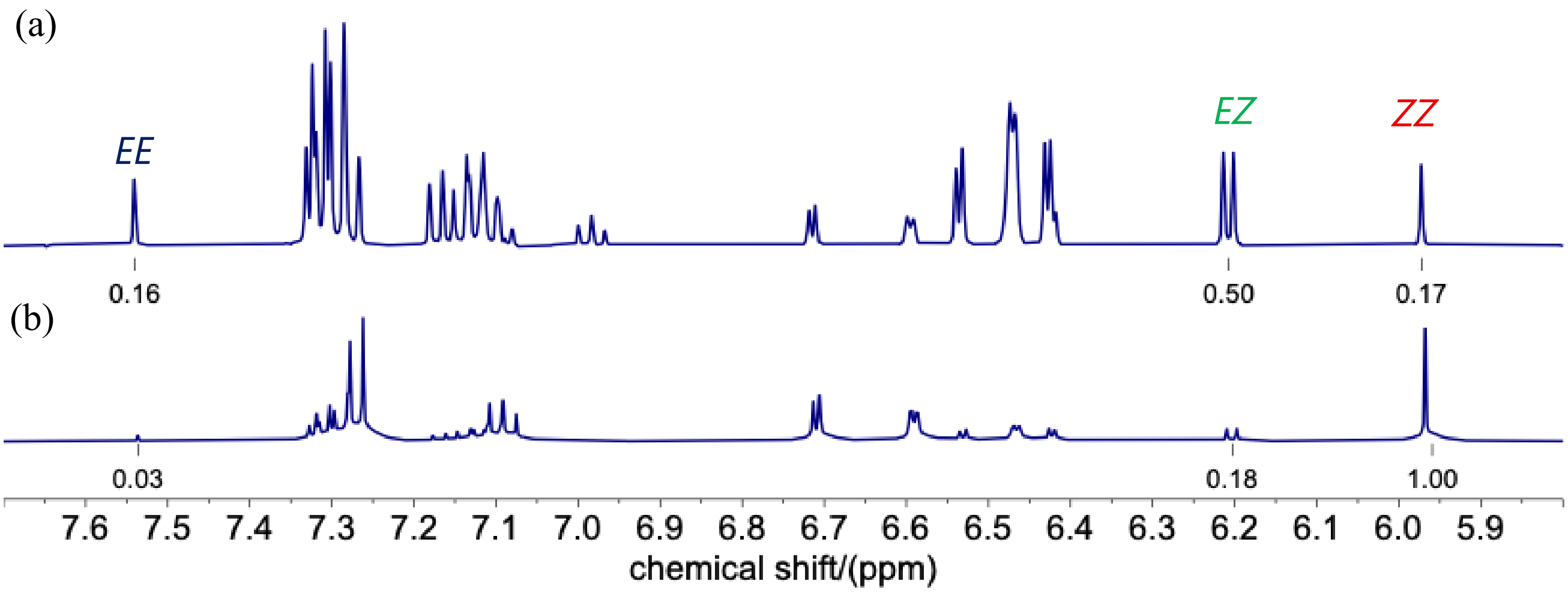

Figure S16. ^{1}H NMR spectra (500 MHz, [D_{12}]cyclohexane) of **1b** (a) the photostationary state (PSS) after 310 nm irradiation and (b) the same sample after heating at 65 °C for 3 days.

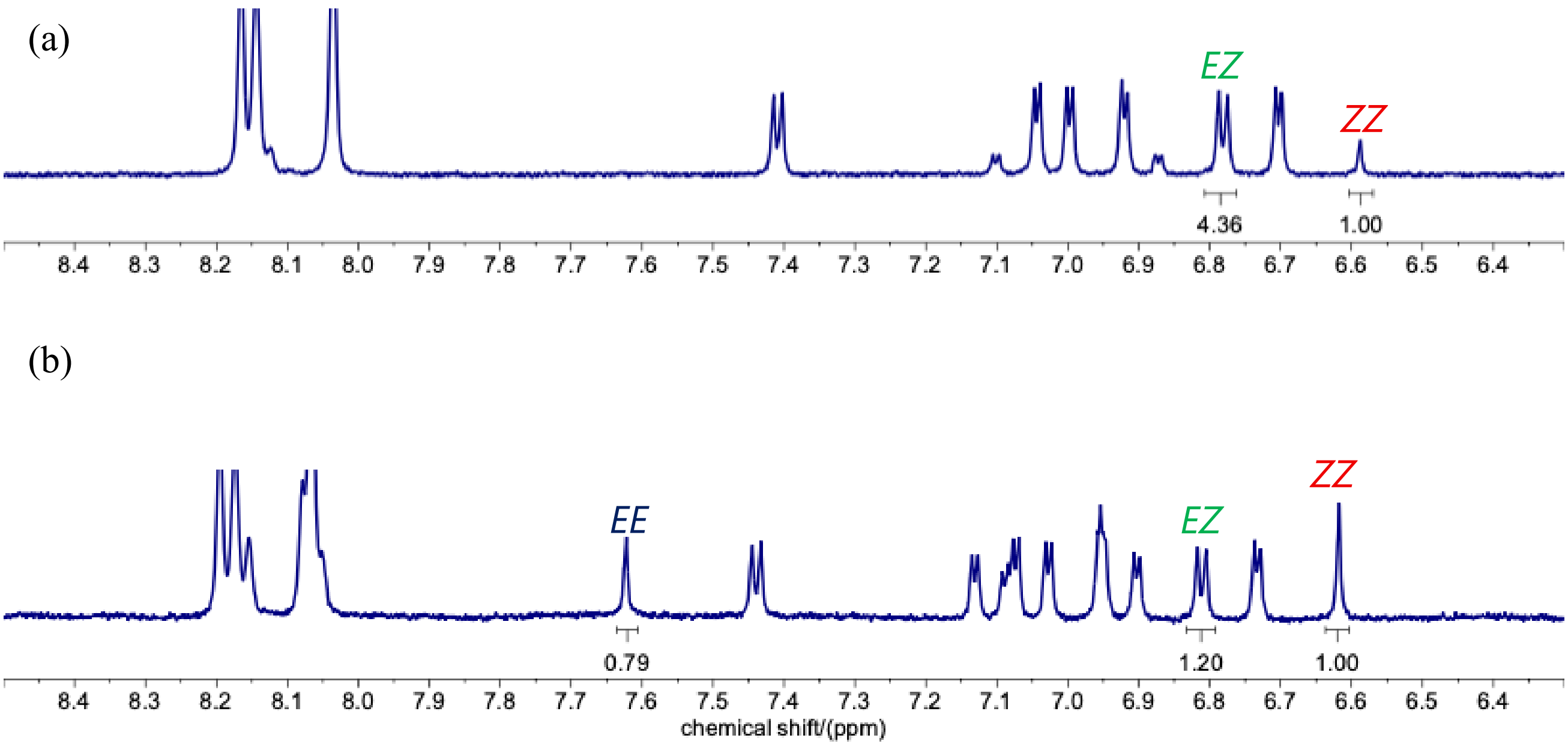

Figure S17. ^{1}H NMR spectra (500 MHz, [D_6]acetone) of **1a** (a) before and (b) after irradiation at 450 nm for 17 min. The observation of the *EE* isomer in the spectrum after irradiation at 450 nm, along with changes in the geometrical isomer ratios, clearly indicates photoisomerization.

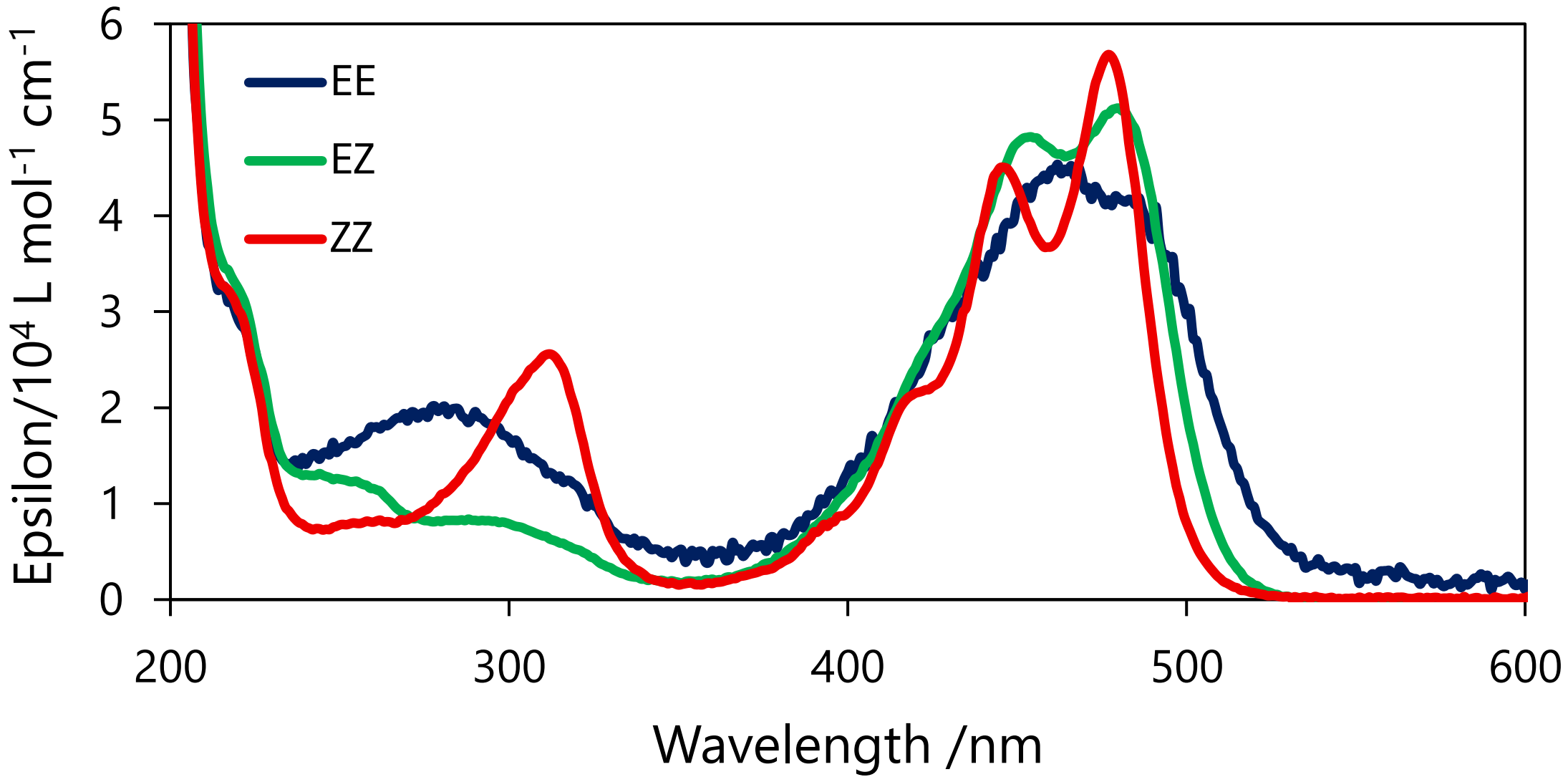

Figure S18. UV–vis absorption spectra of all three individual geometrical isomers (*EE*, *EZ*, and *ZZ*-**1b**) in acetonitrile.

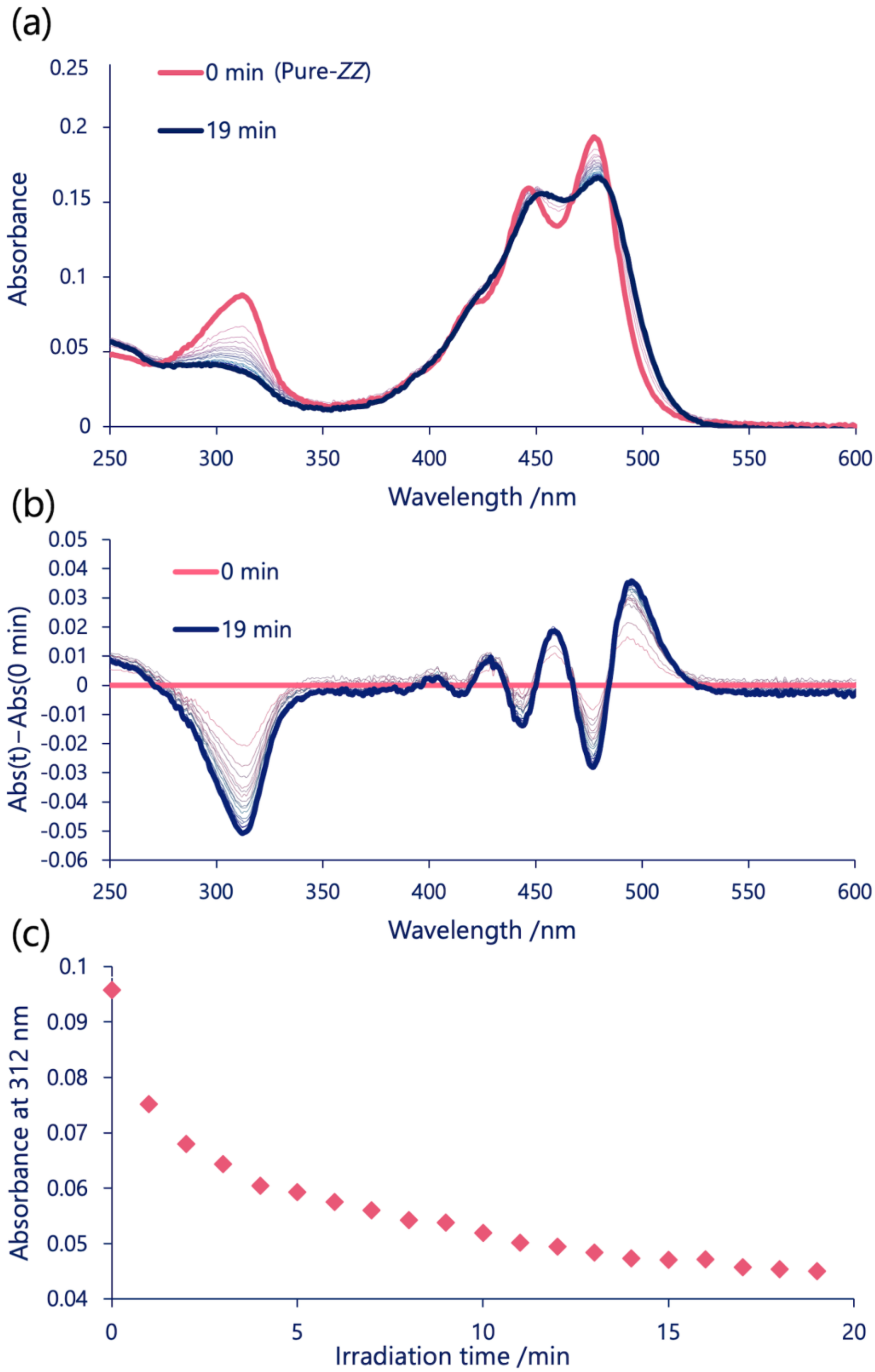

Figure S19. UV–vis absorption spectral changes upon irradiation of *ZZ*-**1b** in acetonitrile at 25 °C with 312 nm light. (a) Absorption spectra recorded at 1 min intervals over a total irradiation period of 19 min. (b) Difference spectra obtained by subtracting the spectrum at 0 min from each subsequent spectrum. (c) Time course of the absorbance at 312 nm.

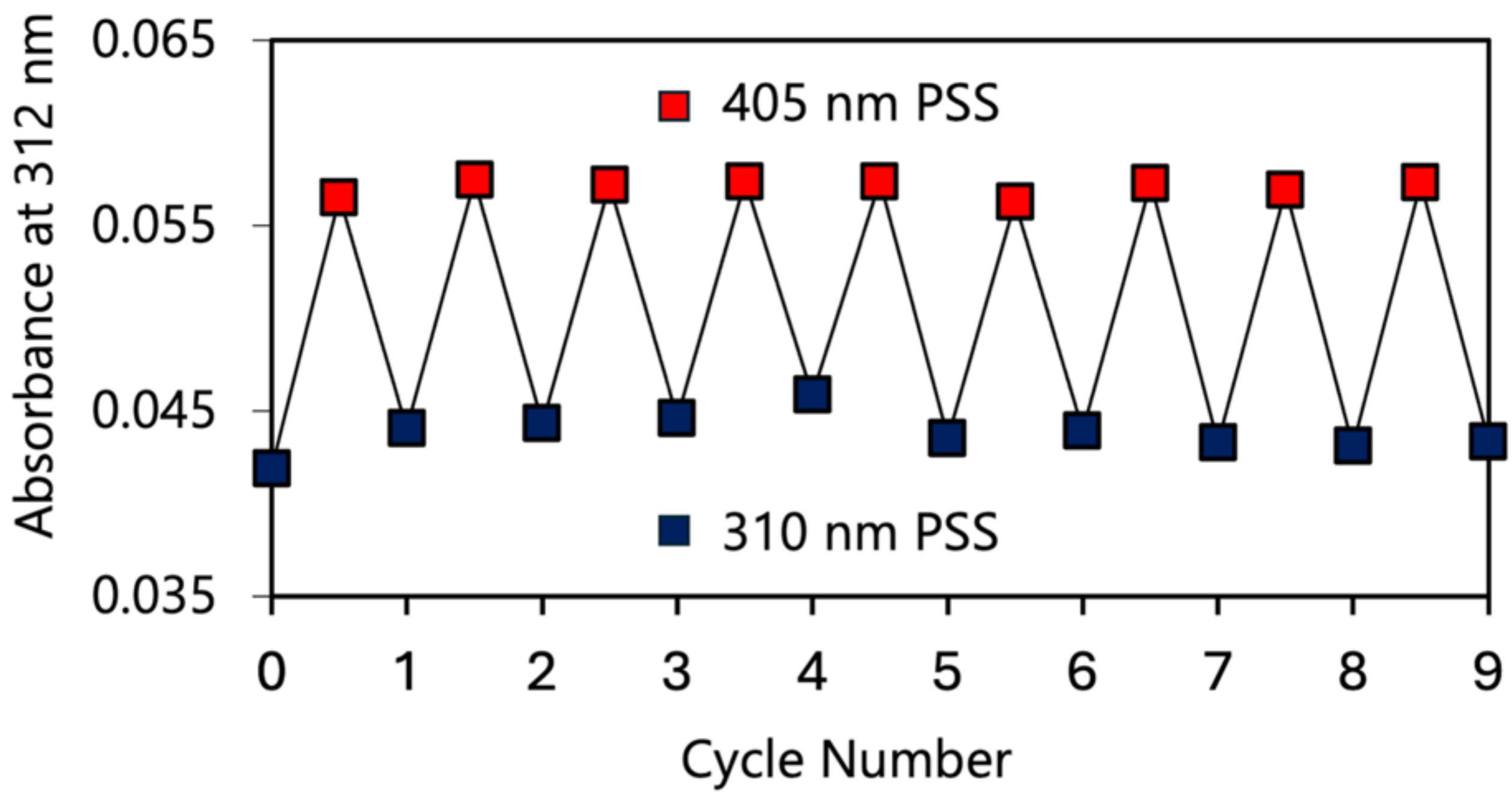

Figure S20. Fatigue resistance of **1b** in cyclohexane over 9 switching cycles. In each cycle, the sample was irradiated at 310 nm to generate a photostationary state enriched in *EZ*-**1b**, followed by irradiation at 405 nm to induce partial back-isomerization.

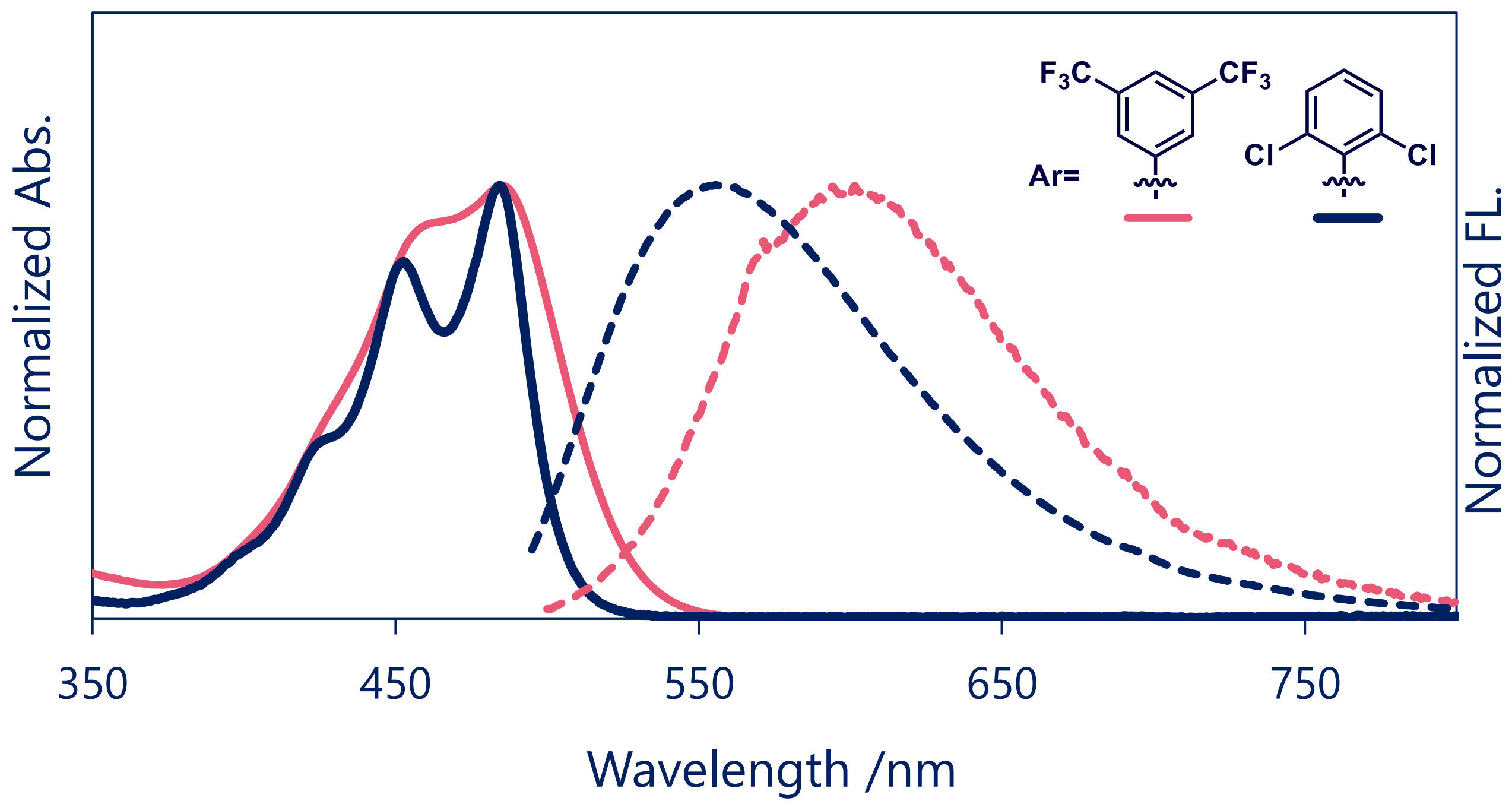

Figure S21. Absorption (solid line) and emission (dashed line) spectra of **1a** and **1b** in cyclohexane.

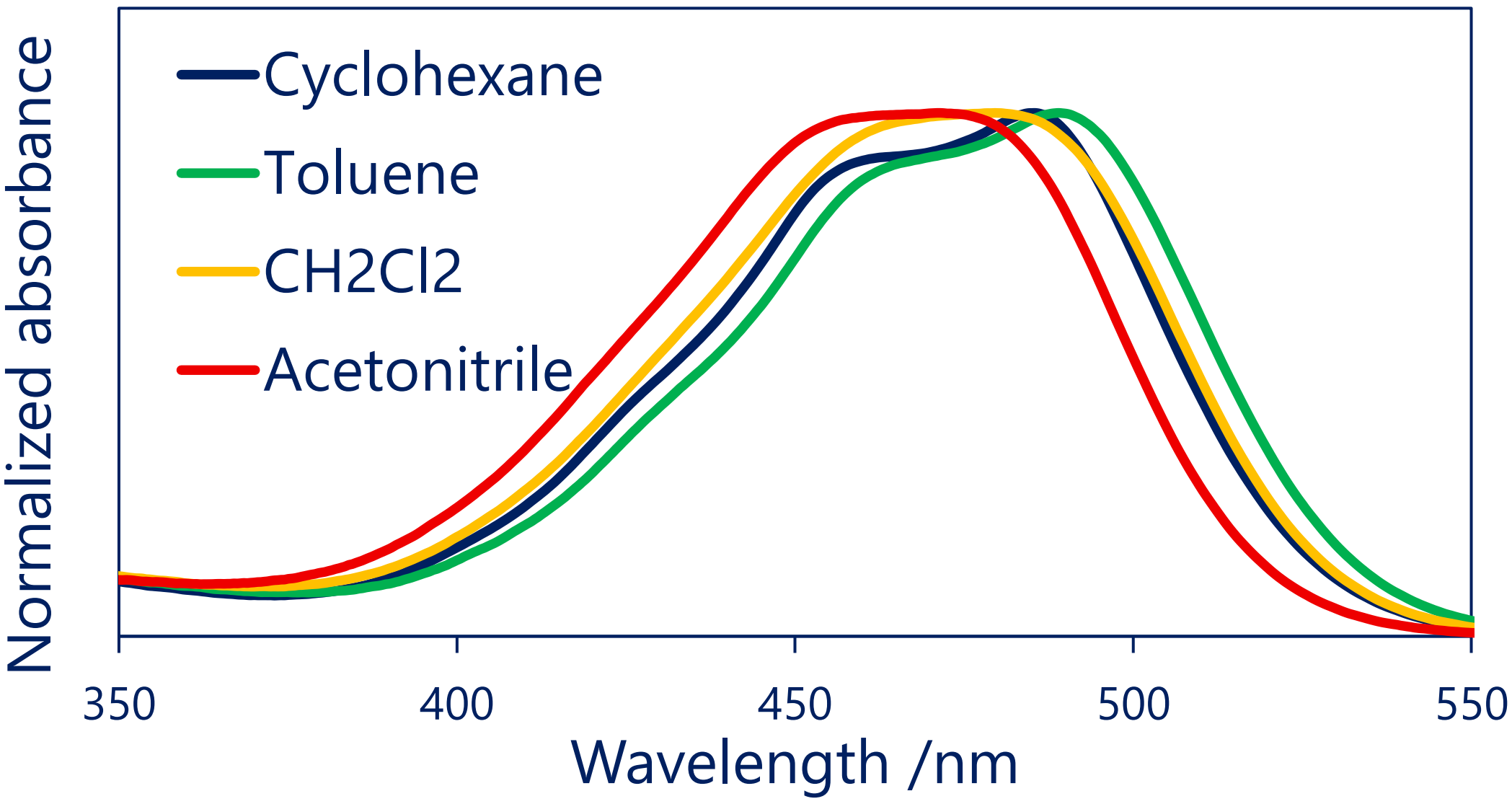

Figure S22. Solvent dependence of the absorption spectra of **1a**.

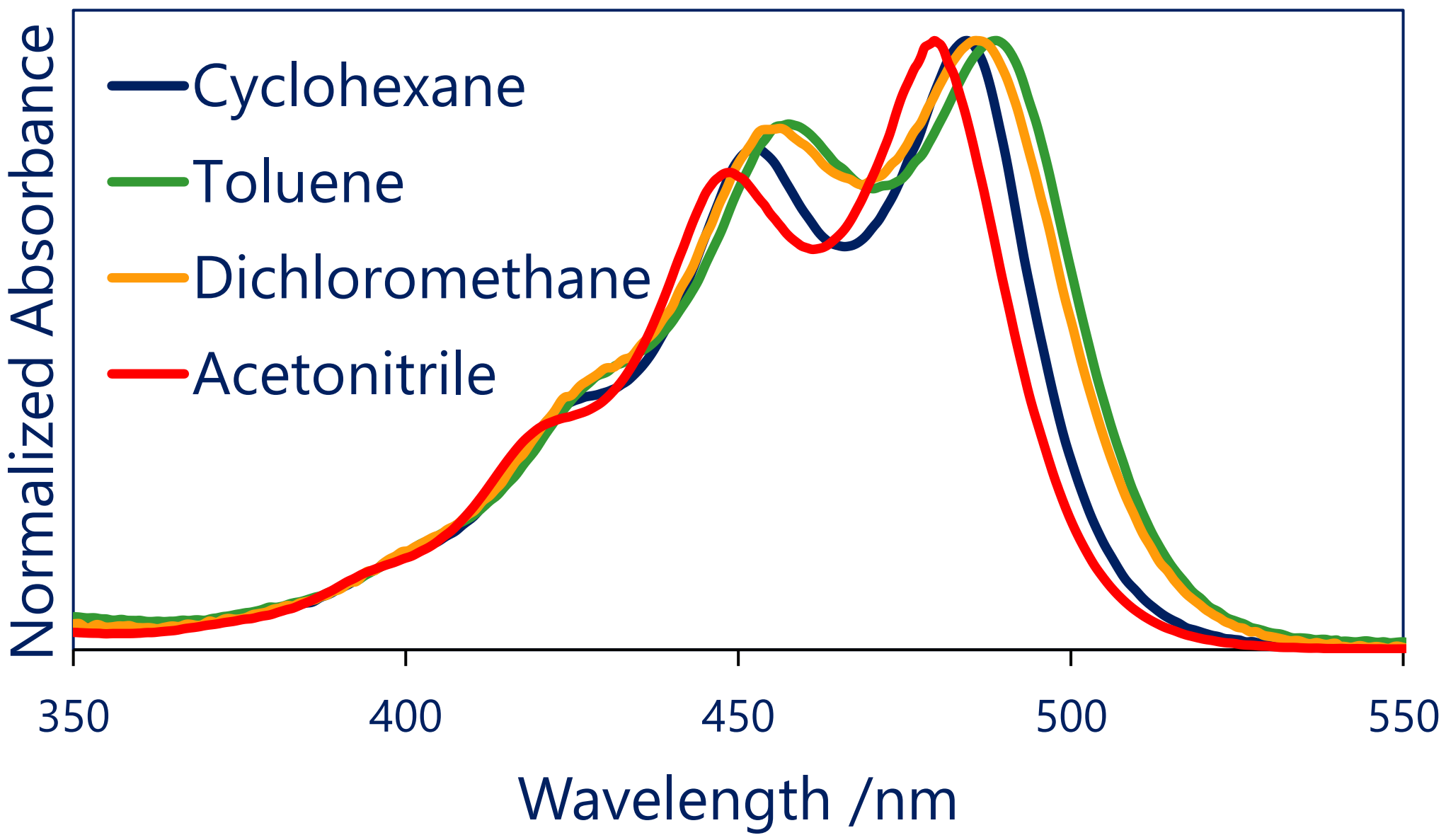

Figure S23. Solvent dependence of the absorption spectra of *ZZ*-**1b**.

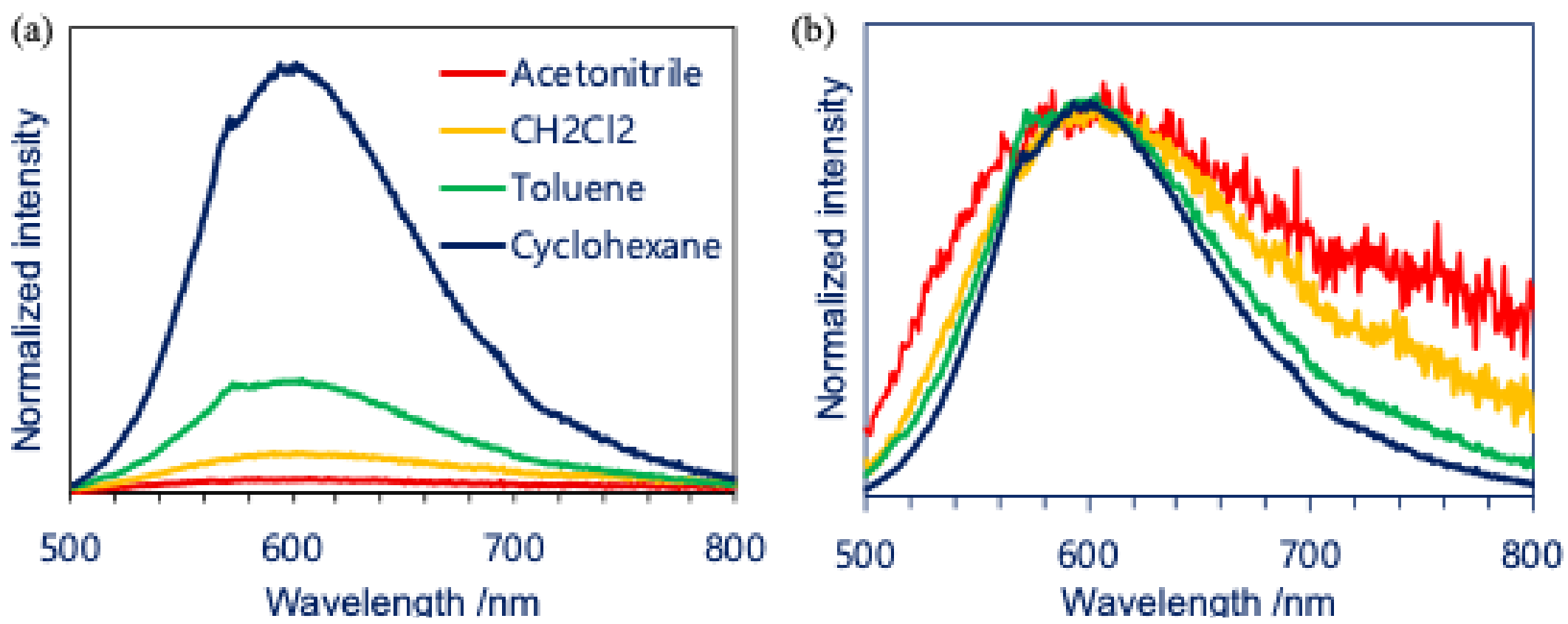

Figure S24. Solvent-dependent emission spectra of **1a** (λ_{ex} = 489 nm). (a) Emission spectra corrected for absorbance at the excitation wavelength so that the relative area underneath the curves reflects the ratio of the fluorescence quantum yields. (b) Emission spectra normalized to the emission maximum of each spectrum.

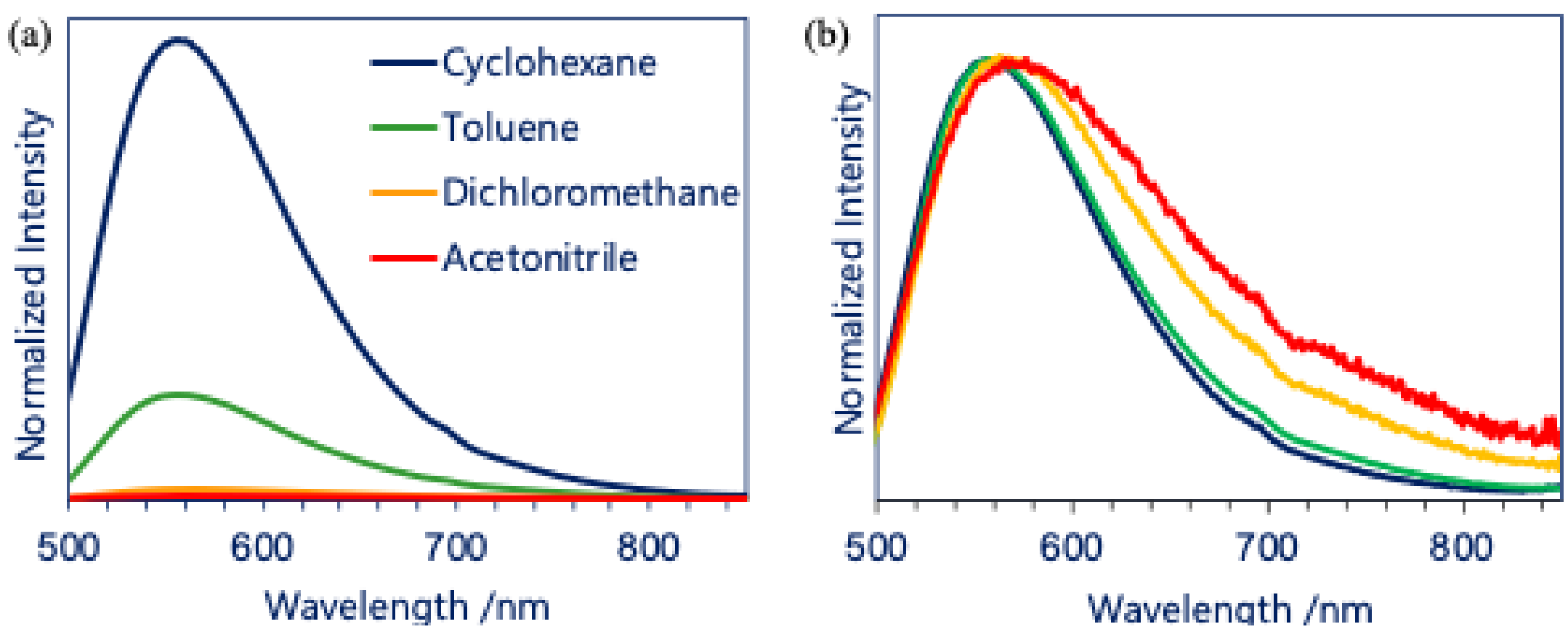

Figure S25. Solvent-dependent emission spectra of *ZZ*-**1b** (λ_{ex} = 450 nm). (a) Emission spectra corrected for absorbance at the excitation wavelength so that the relative area underneath the curves reflects the ratio of the fluorescence quantum yields. (b) Emission spectra normalized to the emission maximum of each spectrum.

Table S2. Fluorescence quantum yield (Φ_{fl}) of *ZZ*-**1b** (λ_{ex} = 450 nm) and **1a** (λ_{ex} = 489 nm) in each solvent.

**Solvent**	Φ_{fl}	
	ZZ-**1b**	**1a**
Cyclohexane	0.40	0.0059
Toluene	0.10	0.0020
Dichloromethane	0.011	0.0009
Acetonitrile	0.005	0.0013

*Determined using a relative method with fluorescein (Φ_{fl} = 0.88 in 0.10 M NaOH aq.) as a standard.

Table S3. Fluorescence lifetime of **1b** in cyclohexane (λ_{ex} = 405 nm)

	τ_1	τ_2	τ_3
ZZ-**1b**	0.23 ns	2.65 ns	
**1b** after 310 nm irradiation	0.15 ns	2.23 ns	3.45 ns

Table S3 summarizes the fluorescence lifetime measurements for *ZZ*-**1b** and the sample after photoirradiation. The measurements were performed at an excitation wavelength of 405 nm, and the lifetimes were monitored at 550 nm. The decay profile of *ZZ*-**1b** was fitted well with a biexponential function, whereas that of the photoirradiated sample required a triexponential function. In both samples, short-lived components and components with lifetimes of approximately 2 ns were observed. By contrast, an additional component with a lifetime of approximately 3 ns emerged in the sample after photoirradiation. These results suggest that the 3 ns component originates from a geometrical isomer other than the *ZZ* form.

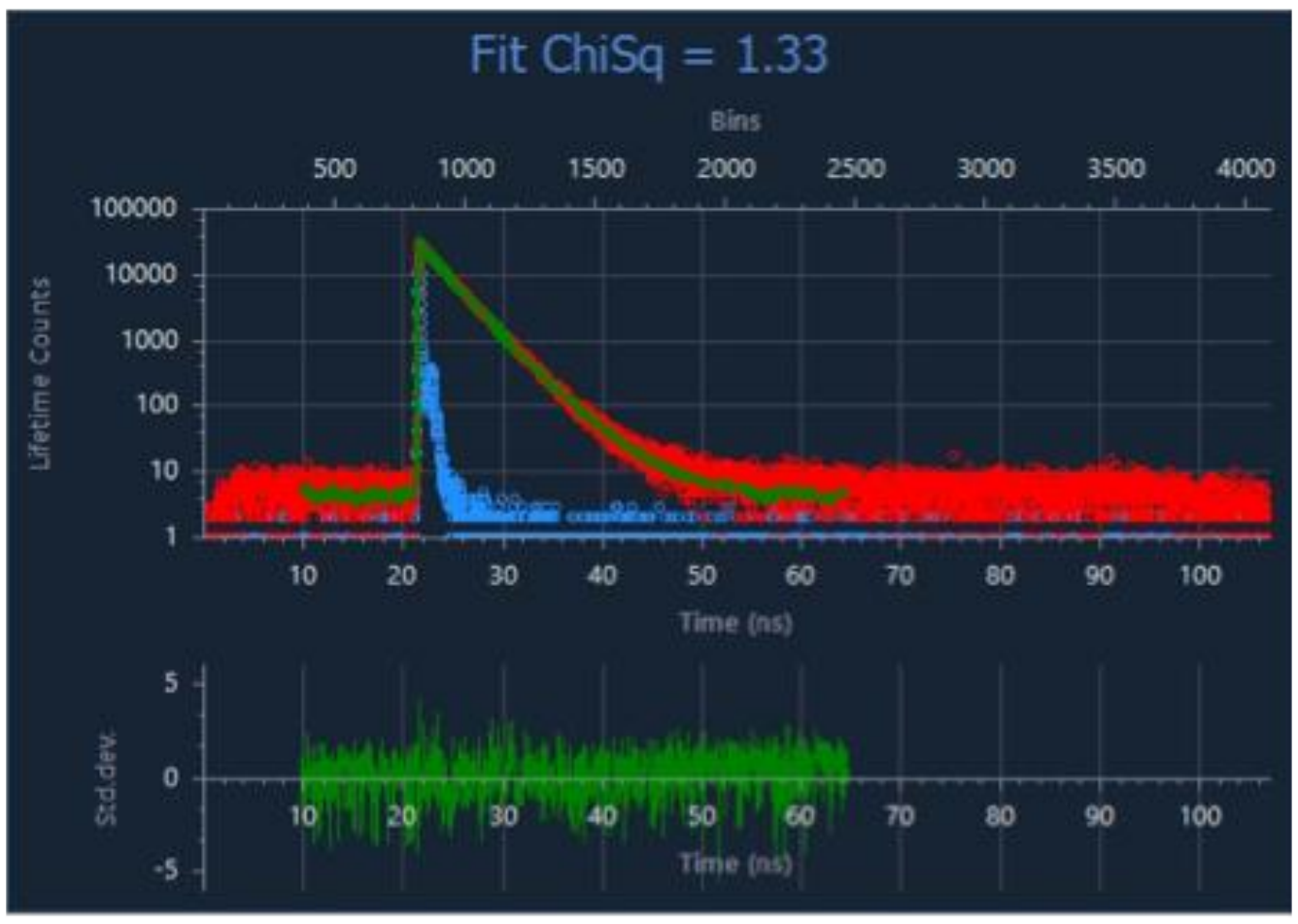

Figure S26. Fluorescence decay curves of *ZZ*-**1b** in cyclohexane (λ_{ex} = 405 nm).

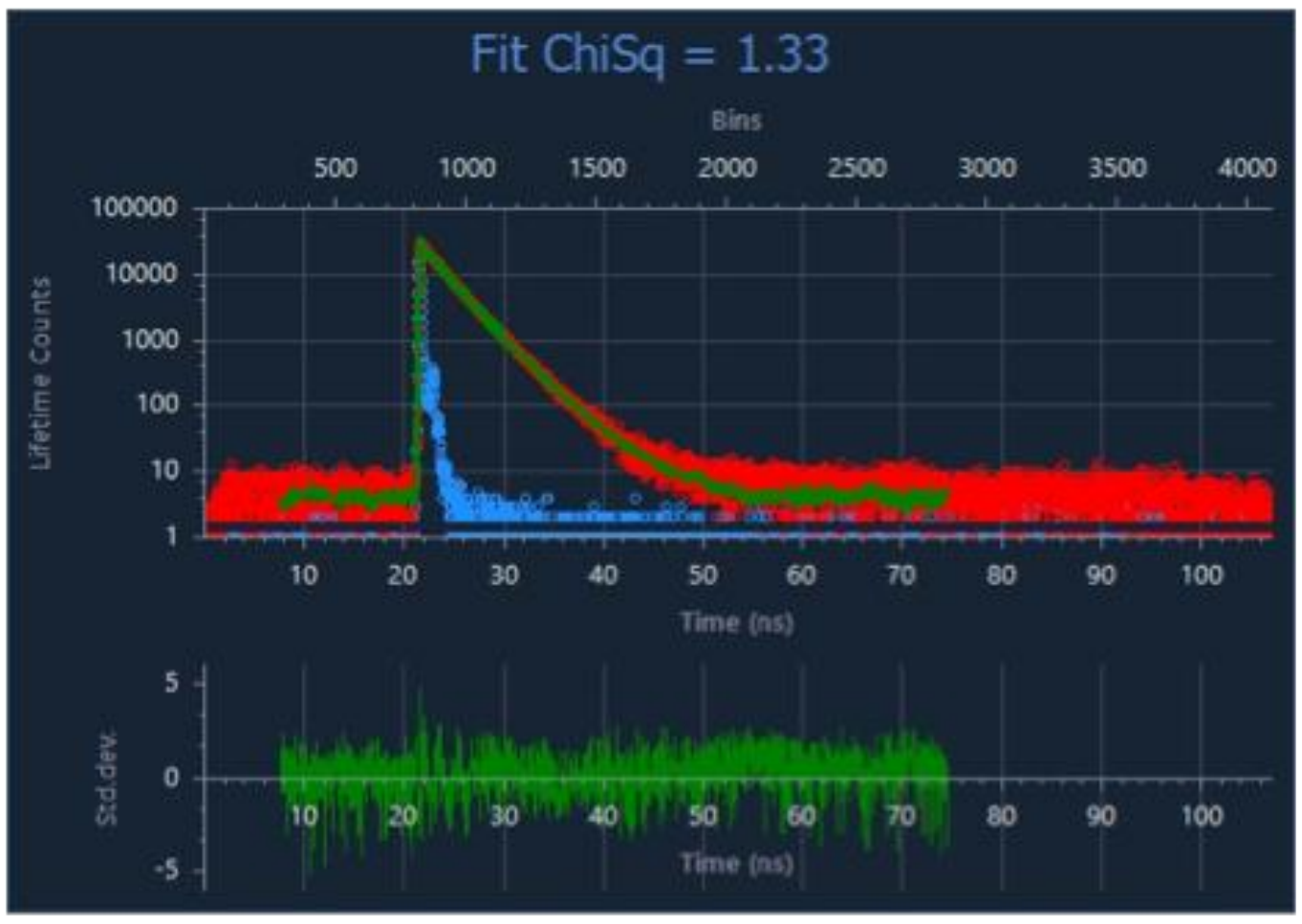

Figure S27. Fluorescence decay curves of **1b** after 310 nm irradiation in cyclohexane (λ_{ex} = 405 nm).

3. X-Ray crystal structure determinations

Brown plate crystals of *EE*-**1b** were grown by slow diffusion of methanol vapor into the chloroform solution of the mixture of the geometrical isomers of **1b**. Single-crystal X-ray diffraction data were collected on a Rigaku XtaLAB Synergy Custom diffractometer using multilayer mirror monochromated Mo-*K*α radiation (λ = 0.71075 Å) by the ω scan mode. The crystal was cooled by a stream of cold N_2 gas. Collection, indexing, peak integration, cell refinement, and scaling of the diffraction data were performed using CrysAlisPro 1.171.40.75a (Rigaku OD, 2020). The data were corrected for Lorentz and polarization effects, and empirical absorption correction was applied. The structures were solved using SHELXT[33] programs and refined by full-matrix least-squares calculations on F^2 (SHELXL).[34,35] All non-hydrogen atoms were modeled anisotropically. All hydrogen atoms were placed in idealized positions and refined using a riding model [U_{iso}(H) = 1.2U_{eq}(C)].

The crystallographic data are summarized in Table S4. Deposition Number 2540931 contains the supplementary crystallographic data for this paper. These data are provided free of charge by the joint Cambridge Crystallographic Data Centre and Fachinformationszentrum Karlsruhe Access Structures service.

Table S4. Crystallographic data.

	EE-**1b**
CCDC No.	2540931
empirical formula	$C_{28}H_{18}Cl_4S_3$
formula weight	592.40
temperature / K	173(2)K
crystal system	Orthorhombic
space group	Pbca
a / Å	20.661(2)
b / Å	12.0047(10)
c / Å	21.218(3)
α / deg	90
β / deg	90
γ / deg	90
V / Å^3	5262.6(11)
Z	8
D (calcd) / g cm^{-3}	1.495
μ / mm^{-1}	0.706
F(000)	2416
crystal size / mm^3	0.100×0.020×0.010
θ range for data collection / deg	1.920 to 30.305
index ranges	$-24 \leq h \leq 25$,
	$-12 \leq k \leq 15$,
	$-28 \leq l \leq 26$
reflections collected	20688
independent reflections	5903 [R(int) = 0.1308]
completeness	94.0%
data / restraints / parameters	5903 / 0 / 318
GOF on F^2	1.056
final R indices [$I > 2\sigma(I)$]^a	R_1 = 0.1075,
	wR_2 = 0.1678
R indices (all data)^a	R_1 = 0. 2318,
	wR_2 = 0.2049
largest diff. peak and hole / e·A^{-3}	0.481 and -0.317

^{a} $R_1 = \sum||F_o|-|F_c||/\sum|F_o|$, $wR_2 = [\sum[w(F_o^2-F_c^2)^2]/\sum w(F_o^2)^2]^{1/2}$.

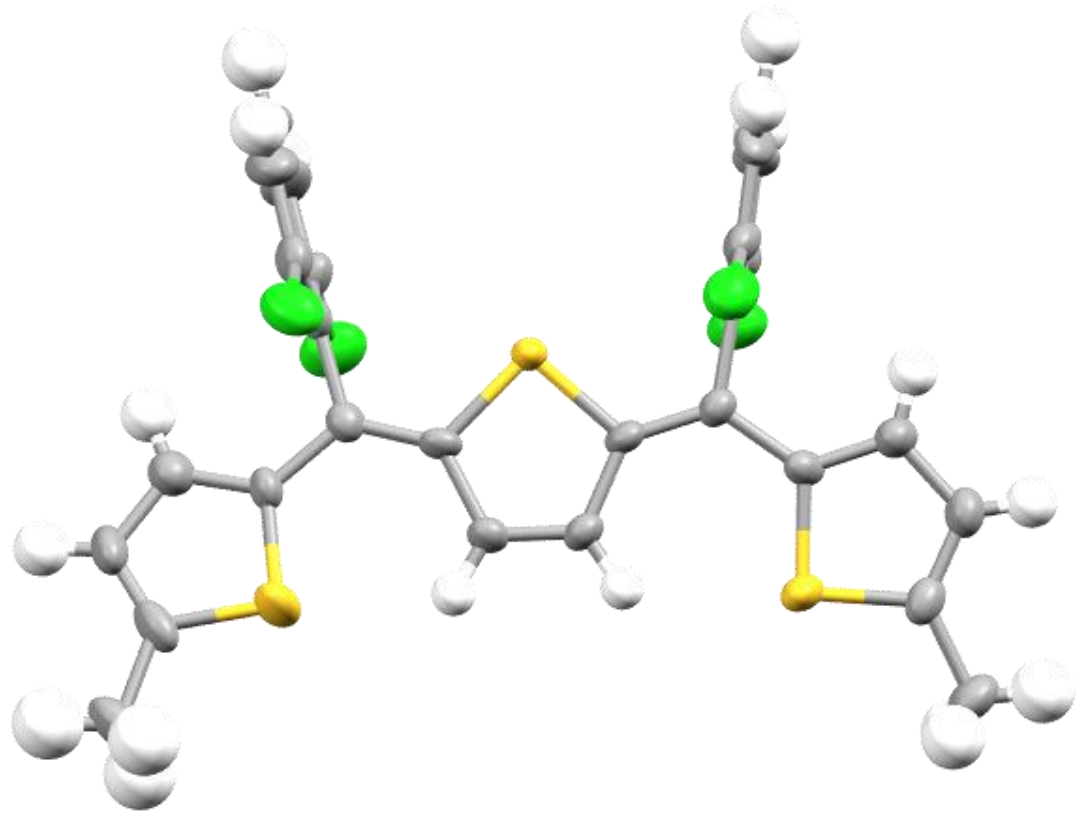

Figure S28. The crystal structure of *EE*-**1b** (C = gray, H = white, S = orange, and Cl = green). Thermal ellipsoids are drawn at 50% probability level.

4. Theoretical calculations

All calculations were carried out using the Gaussian 16 program package (Revision C.01).[36] Geometry optimizations of all isomers of **1b** were performed using density functional theory (DFT) at the CAM-B3LYP-D3BJ/6-311+G(d,p) level with the SMD solvation model using cyclohexane as the solvent. Frequency calculations were carried out at the same level of theory to confirm that the optimized geometries correspond to local minima. Excitation energies and oscillator strengths were computed using time-dependent density functional theory (TD-DFT) at the same level of theory.

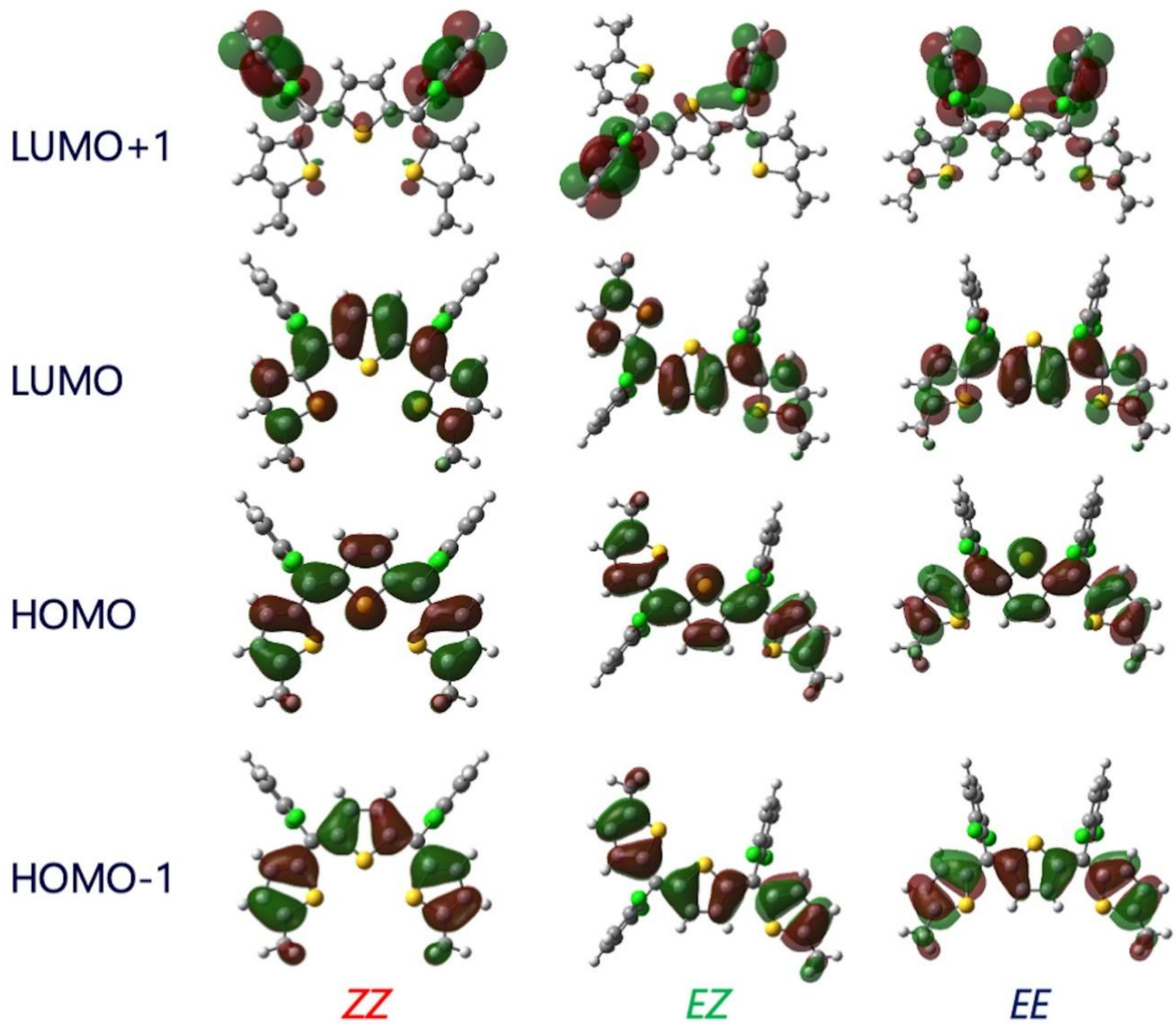

Figure S29. Molecular orbitals (MOs) of **1b** in each isomer.

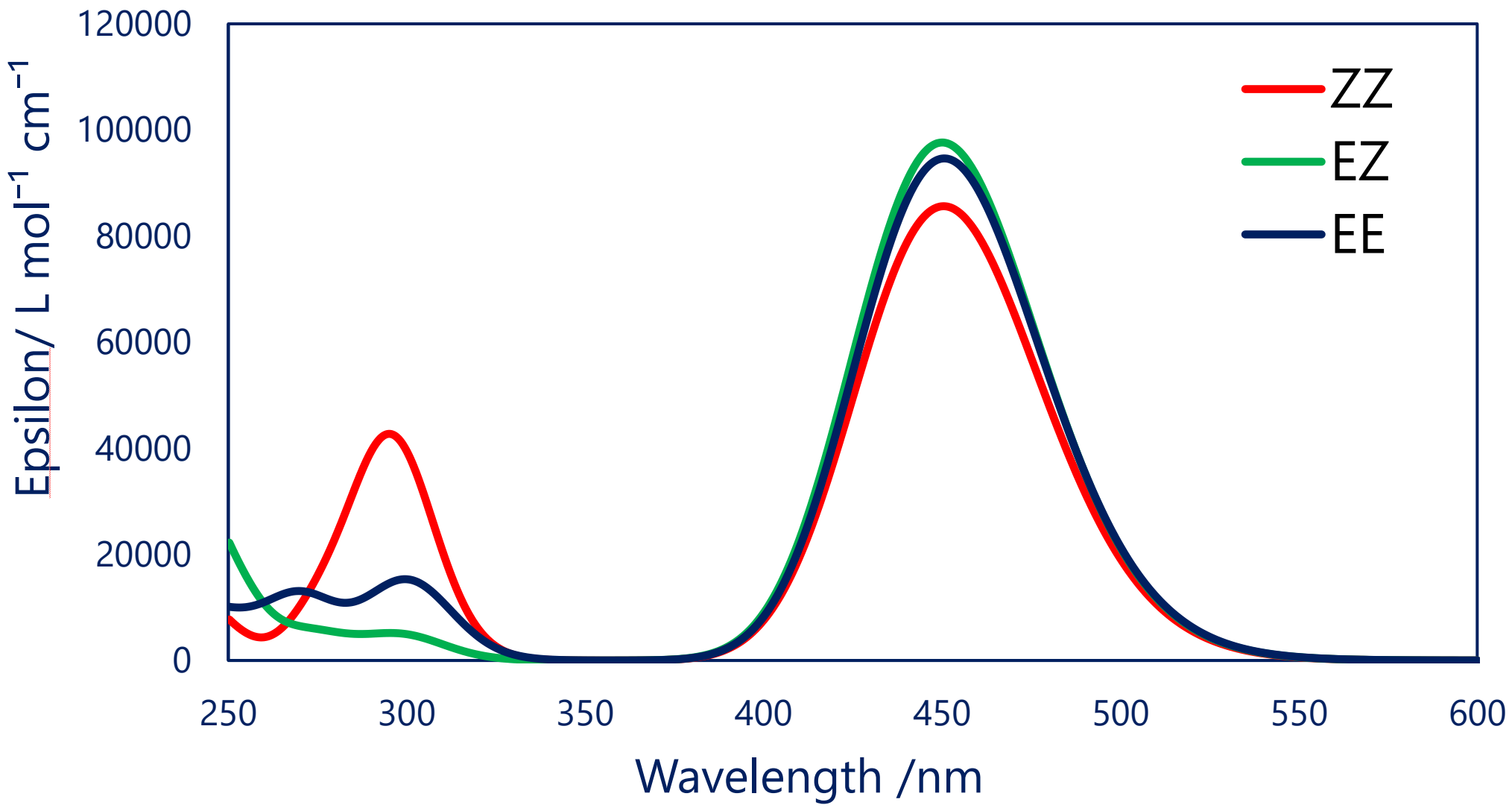

Figure S30. Calculated absorption of each isomer of **1b** in cyclohexane.

Table S5. Summary of the TD-DFT calculation of *EE*-**1b**.

No.	Energy (cm^{-1})	Wavelength (nm)	Osc. strength	Major contributions
1	22201.2172	450.425754	1.3059	HOMO->LUMO (97%)
2	33081.6365	302.282507	0.1314	H-1->LUMO (14%), HOMO->L+1 (74%)
3	33498.6251	298.519714	0.0548	H-1->LUMO (67%), HOMO->L+1 (15%), HOMO->L+5 (11%)
4	34222.1044	292.208798	0.0275	HOMO->L+2 (90%)
5	35846.505	278.967224	0.0234	H-1->LUMO (12%), HOMO->L+5 (79%)
6	36278.8182	275.642937	0.0121	HOMO->L+4 (92%)
7	36308.6607	275.416383	0.0115	HOMO->L+3 (93%)
8	37169.2543	269.039565	0.1253	H-9->LUMO (41%), H-6->LUMO (41%)
9	39172.7355	255.279594	0.0396	H-4->LUMO (41%), H-3->LUMO (36%)
10	39234.8402	254.875512	0.0171	H-5->LUMO (80%)
11	39588.111	252.60109	0.0156	HOMO->L+6 (34%), HOMO->L+7 (22%), HOMO->L+10 (18%)
12	40830.2049	244.916724	0.0416	HOMO->L+13 (21%), HOMO->L+24 (14%), HOMO->L+28 (10%)
13	41207.6723	242.673256	0.0006	HOMO->L+6 (37%), HOMO->L+10 (15%), HOMO->L+11 (25%)
14	41318.1703	242.02427	0	H-2->LUMO (49%), H-2->L+2 (12%)
15	41362.5308	241.764704	0.016	H-4->LUMO (22%), H-3->LUMO (25%)
16	41756.1293	239.485799	0.0395	H-9->LUMO (43%), H-6->LUMO (34%)
17	42036.0037	237.89131	0.0238	H-7->LUMO (84%)
18	42231.9965	236.78729	0.0053	HOMO->L+8 (60%)
19	42748.9978	233.923612	0.0053	H-8->LUMO (77%)
20	42945.7971	232.851656	0.002	H-3->L+1 (10%), H-2->LUMO (39%), H-2->L+2 (15%)
21	43009.5149	232.506691	0.0008	H-4->LUMO (18%), H-3->LUMO (20%), H-2->L+1 (15%)
22	43458.7657	230.103175	0.0191	HOMO->L+7 (29%), HOMO->L+11 (28%)
23	43627.3356	229.214089	0.0031	HOMO->L+7 (26%), HOMO->L+10 (31%), HOMO->L+20 (13%)
24	44731.5086	223.556064	0.0048	HOMO->L+12 (50%), HOMO->L+13 (13%), HOMO->L+17 (12%)
25	44929.921	222.568831	0.0013	HOMO->L+9 (49%)
26	45456.6011	219.990051	0	HOMO->L+13 (10%), HOMO->L+15 (10%), HOMO->L+24 (17%), HOMO->L+32 (12%)
27	45859.0717	218.059364	0.0078	HOMO->L+14 (14%), HOMO->L+20 (34%)
28	46399.4632	215.519735	0.0131	HOMO->L+14 (52%)
29	46463.9875	215.220443	0.0074	H-7->L+1 (10%), H-4->L+4 (11%), H-3->L+4 (17%), H-2->L+3 (28%)
30	46574.4855	214.709833	0.0534	H-8->L+1 (10%), H-7->L+2 (11%), H-4->L+3 (14%), H-3->L+3 (19%), H-2->L+4 (30%)

Table S6. Summary of the TD-DFT calculation of *EZ*-**1b**.

No.	Energy (cm^{-1})	Wavelength (nm)	Osc. strength	Major contributions
1	22230.2532	449.837432	1.3473	HOMO->LUMO (97%)
2	33200.2	301.203005	0.0098	HOMO->L+1 (67%), HOMO->L+3 (13%)
3	33476.8481	298.713904	0.051	H-1->LUMO (76%)
4	34532.6279	289.581205	0.0059	HOMO->L+1 (15%), HOMO->L+3 (72%)
5	35709.3908	280.038381	0.0057	HOMO->L+2 (12%), HOMO->L+5 (58%)
6	36102.1828	276.991562	0.0141	HOMO->L+2 (24%), HOMO->L+4 (69%)
7	36533.6894	273.71996	0.0053	HOMO->L+2 (52%), HOMO->L+4 (17%), HOMO->L+5 (17%)
8	36724.8428	272.295243	0.042	H-9->LUMO (17%), H-6->LUMO (15%), H-2->LUMO (47%)
9	37724.1638	265.082085	0.0067	HOMO->L+6 (16%), HOMO->L+10 (15%), HOMO->L+12 (13%), HOMO->L+15 (13%)
10	39217.096	254.990834	0.0587	H-4->LUMO (80%)
11	39930.0901	250.437702	0.0831	H-9->LUMO (12%), H-6->LUMO (28%), H-2->LUMO (22%)
12	40280.9413	248.256363	0.0151	HOMO->L+6 (48%)
13	40926.9914	244.33753	0.0987	HOMO->L+10 (24%)
14	41329.4621	241.958146	0.0094	H-7->L+4 (11%), H-3->LUMO (54%), H-3->L+1 (10%), H-3->L+3 (11%)
15	41931.9582	238.481588	0.0088	H-8->LUMO (26%), H-8->L+2 (24%), H-5->L+1 (22%), H-5->L+3 (17%)
16	42079.5577	237.645084	0.1523	H-7->LUMO (43%)
17	42243.2882	236.723996	0.001	H-5->LUMO (88%)
18	42347.3337	236.142376	0.0497	H-7->LUMO (37%), HOMO->L+7 (12%), HOMO->L+12 (13%)
19	42685.28	234.272798	0.1743	H-9->LUMO (34%), H-6->LUMO (26%)
20	42763.5158	233.844197	0.0138	H-7->L+4 (17%), H-3->LUMO (32%), H-3->L+1 (15%), H-3->L+3 (16%)
21	42847.3975	233.386404	0.0387	H-8->LUMO (57%)
22	43292.6155	230.986275	0.0037	HOMO->L+9 (30%), HOMO->L+11 (20%)
23	43970.9278	227.422993	0.002	HOMO->L+7 (32%), HOMO->L+8 (27%), HOMO->L+20 (11%)
24	44324.1986	225.610396	0.0011	HOMO->L+9 (10%), HOMO->L+11 (30%), HOMO->L+12 (10%), HOMO->L+13 (14%)
25	44545.1946	224.491106	0.0028	HOMO->L+9 (22%), HOMO->L+12 (10%), HOMO->L+13 (21%)
26	44850.8787	222.961072	0.0036	HOMO->L+10 (31%), HOMO->L+13 (12%), HOMO->L+14 (18%)
27	46443.8237	215.313883	0.0178	H-3->L+4 (23%), HOMO->L+11 (10%)
28	46518.0267	214.970426	0.0221	H-3->L+4 (29%), HOMO->L+14 (12%)
29	46610.7805	214.542642	0.0114	HOMO->L+14 (17%)
30	46622.8788	214.48697	0.0262	H-8->L+1 (13%), H-5->L+2 (60%)

Table S7. Summary of the TD-DFT calculation of *ZZ*-**1b**.

No.	Energy (cm^{-1})	Wavelength (nm)	Osc. strength	Major contributions
1	22210.0893	450.245826	1.1816	HOMO->LUMO (97%)
2	33759.1422	296.216057	0.5479	H-1->LUMO (90%)
3	33918.0334	294.828414	0.0001	HOMO->L+1 (92%)
4	33962.3939	294.44332	0.0018	HOMO->L+3 (80%)
5	35829.5674	279.0991	0.1168	HOMO->L+2 (46%), HOMO->L+5 (43%)
6	36015.0749	277.661508	0.0005	H-9->LUMO (14%), H-6->LUMO (10%), H-2->LUMO (71%)
7	36374.7982	274.915615	0.0117	HOMO->L+4 (94%)
8	36602.2465	273.207274	0.0317	HOMO->L+2 (45%), HOMO->L+5 (42%)
9	36757.9115	272.050277	0.0071	HOMO->L+3 (11%), HOMO->L+12 (51%), HOMO->L+13 (10%)
10	39400.9904	253.800727	0.002	H-3->LUMO (86%)
11	39507.4556	253.116781	0.0074	HOMO->L+6 (80%)
12	40364.823	247.740465	0.0623	H-9->LUMO (16%), H-6->LUMO (49%), H-2->LUMO (15%)
13	41648.8576	240.102624	0.0022	HOMO->L+10 (65%)
14	41908.5681	238.61469	0.002	H-8->L+2 (13%), H-7->LUMO (32%), H-7->L+4 (12%), H-5->L+3 (19%), H-4->L+1 (20%)
15	42006.9678	238.055745	0.0001	H-8->LUMO (15%), H-8->L+4 (14%), H-7->L+2 (16%), H-5->L+1 (25%), H-4->L+3 (25%)
16	42100.5281	237.526712	0.114	HOMO->L+14 (53%), HOMO->L+15 (13%)
17	42345.7206	236.151371	0.0002	H-4->LUMO (94%)
18	42364.2714	236.047964	0.0045	H-5->LUMO (95%)
19	42643.3392	234.503212	0	HOMO->L+9 (72%)
20	42715.1226	234.109126	0.0123	H-7->LUMO (62%), H-5->L+3 (10%), H-4->L+1 (11%)
21	43078.0721	232.136665	0.0001	H-8->LUMO (79%)
22	43362.7858	230.61249	0.0026	HOMO->L+7 (69%)
23	43407.9528	230.372532	0.1116	H-9->LUMO (50%), H-6->LUMO (30%)
24	44102.3962	226.745049	0	HOMO->L+8 (64%), HOMO->L+13 (11%)
25	44214.5072	226.170111	0.0036	HOMO->L+11 (66%), HOMO->L+18 (15%)
26	45128.3334	221.590279	0.0004	H-1->L+12 (13%), HOMO->L+10 (16%), HOMO->L+15 (23%), HOMO->L+21 (20%)
27	46154.2706	216.664674	0.0048	HOMO->L+15 (17%), HOMO->L+17 (19%), HOMO->L+21 (35%)
28	46321.2274	215.883744	0	HOMO->L+8 (17%), HOMO->L+12 (10%), HOMO->L+13 (46%)
29	46592.2297	214.628063	0.0353	H-8->L+3 (12%), H-7->L+1 (13%), H-5->L+2 (34%), H-4->L+4 (36%)
30	46632.5574	214.442453	0.0434	H-8->L+1 (12%), H-7->L+3 (13%), H-5->L+4 (36%), H-4->L+2 (34%)

Table S8. Cartesian coordinates for the optimized structure of *EE*-**1b**.

Stoichiometry: $C_{28}H_{18}S_3Cl_4$, full point group: C2, charge: 0, multiplicity: 1.

Total energy: -4113.0608 a.u., No. of imaginary freq: 0.

Center No.	Atomic No.	Coordinates (Angstroms)		
		X	Y	Z
1	6	-0.09281	1.277347	-0.627632
2	6	-0.049619	0.668948	-1.942341
3	6	0.049619	-0.668948	-1.942341
4	6	0.09281	-1.277347	-0.627632
5	16	0	0	0.599549
6	1	0.076539	-1.256664	-2.846989
7	6	-0.183691	2.577614	-0.27423
8	6	0.183691	-2.577614	-0.27423
9	6	-0.362602	3.720517	-1.158974
10	6	-0.063387	5.020932	-0.856416
11	6	-0.404232	5.940431	-1.883589
12	1	0.390879	5.313183	0.080011
13	1	-0.228205	7.006089	-1.818015
14	6	0.362602	-3.720517	-1.158974
15	6	0.063387	-5.020932	-0.856416
16	6	0.404232	-5.940431	-1.883589
17	1	-0.390879	-5.313183	0.080011
18	1	0.228205	-7.006089	-1.818015
19	6	0.112213	-2.912818	1.176937
20	6	1.2692	-3.118935	1.930031
21	6	-1.104017	-3.032464	1.850554
22	6	1.228906	-3.41609	3.281113
23	6	-1.173153	-3.331003	3.201504
24	6	0	-3.520666	3.912573
25	1	-0.043386	-3.751742	4.969471
26	6	-0.112213	2.912818	1.176937
27	6	-1.2692	3.118935	1.930031
28	6	1.104017	3.032464	1.850554
29	6	-1.228906	3.41609	3.281113
30	6	1.173153	3.331003	3.201504

31	6	0	3.520666	3.912573
32	1	0.043386	3.751742	4.969471
33	1	-0.076539	1.256664	-2.846989
34	1	2.150829	-3.560107	3.827872
35	1	-2.13715	-3.413013	3.684785
36	1	2.13715	3.413013	3.684785
37	1	-2.150829	3.560107	3.827872
38	17	2.826398	-2.983533	1.15418
39	17	-2.599433	-2.80991	0.976608
40	17	2.599433	2.80991	0.976608
41	17	-2.826398	2.983533	1.15418
42	16	-1.113281	3.642234	-2.733876
43	16	1.113281	-3.642234	-2.733876
44	6	-1.462243	5.998632	-4.22643
45	1	-2.528046	5.81984	-4.388949
46	1	-0.925902	5.624702	-5.102197
47	1	-1.306208	7.076695	-4.171506
48	6	1.462243	-5.998632	-4.22643
49	1	2.528046	-5.81984	-4.388949
50	1	0.925902	-5.624702	-5.102197
51	1	1.306208	-7.076695	-4.171506
52	6	-0.979778	5.356387	-2.968477
53	6	0.979778	-5.356387	-2.968477

Table S9. Cartesian coordinates for the optimized structure of *EZ*-**1b**.

Stoichiometry: $C_{28}H_{18}S_3Cl_4$, full point group: C1, charge: 0, multiplicity: 1.

Total energy: -4113.0617 a.u., No. of imaginary freq: 0.

Center No.	Atomic No.	Coordinates (Angstroms)		
		X	Y	Z
1	6	1.311526	-0.487341	0.12795
2	6	0.698807	-1.794646	0.232807
3	6	-0.641285	-1.786346	0.191465
4	6	-1.249344	-0.477	0.048165
5	16	0.033173	0.739459	-0.015418
6	1	-1.247301	-2.677611	0.271129
7	6	2.613652	-0.129158	0.136428
8	6	-2.580422	-0.241447	-0.013478
9	6	3.768288	-1.016014	0.149396
10	6	5.038549	-0.670364	0.52339
11	6	5.983811	-1.719851	0.37694
12	1	5.290921	0.312849	0.895087
13	1	7.030541	-1.625142	0.634426
14	6	-3.263893	1.030156	-0.148799
15	6	-4.624395	1.209593	-0.176647
16	6	-5.019583	2.562073	-0.320105
17	1	-5.324503	0.390471	-0.095829
18	1	-6.05143	2.885545	-0.359692
19	6	-3.490211	-1.422756	0.068323
20	6	-3.919084	-2.097433	-1.074839
21	6	-3.97189	-1.892489	1.290335
22	6	-4.775834	-3.184306	-1.014812
23	6	-4.829617	-2.976638	1.380084
24	6	-5.228994	-3.619438	0.219834
25	1	-5.899221	-4.467776	0.278258
26	6	2.939865	1.325721	0.118517
27	6	3.251698	1.984484	-1.071805
28	6	2.947333	2.095806	1.282268
29	6	3.543912	3.336686	-1.113732
30	6	3.236925	3.450265	1.268768

31	6	3.533804	4.065906	0.064326
32	1	3.758913	5.124758	0.043142
33	1	1.280718	-2.693124	0.367289
34	1	-5.080881	-3.680259	-1.926254
35	1	-5.177283	-3.309259	2.348696
36	1	3.228135	4.010819	2.193521
37	1	3.772468	3.808948	-2.059467
38	17	-3.360857	-1.568075	-2.642054
39	17	-3.482323	-1.09939	2.766803
40	17	2.589664	1.342953	2.816811
41	17	3.261155	1.084749	-2.567079
42	16	3.754753	-2.664693	-0.426913
43	16	-2.467555	2.578277	-0.308494
44	6	-4.004548	4.913449	-0.558761
45	1	-3.49766	5.236063	-1.471605
46	1	-3.523178	5.416954	0.283395
47	1	-5.038955	5.255592	-0.609005
48	6	6.131832	-4.164066	-0.408252
49	1	6.046813	-4.442776	-1.461523
50	1	5.71114	-4.97945	0.185548
51	1	7.192197	-4.083545	-0.165873
52	6	-3.973273	3.429199	-0.404944
53	6	5.451166	-2.866672	-0.123523

Table S10. Cartesian coordinates for the optimized structure of *ZZ*-**1b**.

Stoichiometry: $C_{28}H_{18}S_3Cl_4$, full point group: C1, charge: 0, multiplicity: 1.

Total energy: -4113.0625 a.u., No. of imaginary freq: 0.

Center No.	Atomic No.	Coordinates (Angstroms)		
		X	Y	Z
1	6	-1.281186	-0.356122	0.001459
2	6	-0.670023	-1.670529	0.000954
3	6	0.670028	-1.670529	-0.000958
4	6	1.281191	-0.356122	-0.001485
5	16	0.000002	0.867432	-0.000034
6	1	1.271379	-2.568247	-0.001421
7	6	-2.611601	-0.109639	0.003431
8	6	2.611605	-0.109635	-0.00345
9	6	-3.292209	1.170837	-0.00059
10	6	-4.652395	1.354273	0.018181
11	6	-5.045965	2.714771	0.005657
12	1	-5.353483	0.532326	0.040425
13	1	-6.077336	3.041918	0.017784
14	6	3.292204	1.170846	0.000542
15	6	4.652387	1.354294	-0.018275
16	6	5.045945	2.714795	-0.005691
17	1	5.353483	0.532354	-0.040536
18	1	6.077314	3.041951	-0.017787
19	6	3.523547	-1.292138	-0.011058
20	6	3.986835	-1.866871	1.172473
21	6	3.969728	-1.865417	-1.2019
22	6	4.84271	-2.956058	1.180659
23	6	4.825456	-2.954595	-1.223614
24	6	5.259641	-3.49637	-0.02486
25	1	5.928615	-4.347714	-0.030225
26	6	-3.523537	-1.292146	0.011072
27	6	-3.98684	-1.866901	-1.172442
28	6	-3.969697	-1.865408	1.20193
29	6	-4.84271	-2.956093	-1.180597
30	6	-4.825419	-2.95459	1.223676

31	6	-5.259619	-3.496387	0.024937
32	1	-5.92859	-4.347734	0.030327
33	1	-1.271373	-2.568247	0.001438
34	1	5.174857	-3.371683	2.122288
35	1	5.144332	-3.368991	-2.170375
36	1	-5.144277	-3.368972	2.170449
37	1	-5.17487	-3.371735	-2.122214
38	17	3.475233	-1.204203	2.704398
39	17	3.437327	-1.200302	-2.725749
40	17	-3.437275	-1.200266	2.725761
41	17	-3.475267	-1.204254	-2.704386
42	16	-2.494229	2.726153	-0.036254
43	16	2.494207	2.726155	0.03611
44	6	-4.02684	5.076446	-0.043558
45	1	-3.542162	5.477461	-0.937215
46	1	-3.520823	5.501348	0.826993
47	1	-5.060401	5.424727	-0.035722
48	6	4.026802	5.076457	0.043736
49	1	3.542088	5.477393	0.93741
50	1	3.520813	5.501429	-0.826797
51	1	5.060361	5.424747	0.035969
52	6	-3.998656	3.584349	-0.023588
53	6	3.99863	3.584362	0.023633
